# KMT2C mediates the estrogen dependence of breast cancer through regulation of ERα enhancer function

**DOI:** 10.1038/s41388-018-0273-5

**Published:** 2018-05-14

**Authors:** Kinisha Gala, Qing Li, Amit Sinha, Pedram Razavi, Madeline Dorso, Francisco Sanchez-Vega, Young Rock Chung, Ronald Hendrickson, James J. Hsieh, Michael Berger, Nikolaus Schultz, Alessandro Pastore, Omar Abdel-Wahab, Sarat Chandarlapaty

**Affiliations:** 1 Louis V. Gerstner, Jr. Graduate School of Biomedical Sciences, Sloan Kettering Institute, Memorial Sloan Kettering Cancer Center, New York, USA; 2 Human Oncology and Pathogenesis Program, Memorial Sloan Kettering Cancer Center, New York, USA; 3 Basepair, Inc, New York, USA; 4 Department of Medicine, Washington University School of Medicine, St. Louis, MO USA; 5 Microchemistry and Proteomics Core Facility, Memorial Sloan Kettering Cancer Center, New York, USA; 6 Computational Biology Program, Memorial Sloan Kettering Cancer Center, New York, USA; 7 Weill Cornell Medical College, New York, USA

## Abstract

Estrogen receptor alpha (ERα) is a ligand-activated nuclear receptor that directs proliferation and differentiation in selected cancer cell types including mammary-derived carcinomas. These master-regulatory functions of ERα require trans-acting elements such as the pioneer factor FOXA1 to establish a genomic landscape conducive to ERα control. Here, we identify the H3K4 methyltransferase KMT2C as necessary for hormone-driven ERα activity and breast cancer proliferation. KMT2C knockdown suppresses estrogen-dependent gene expression and causes H3K4me1 and H3K27ac loss selectively at ERα enhancers. Correspondingly, KMT2C loss impairs estrogen-driven breast cancer proliferation but has no effect on ER- breast cells. Whereas KMT2C loss disrupts estrogen-driven proliferation, it conversely promotes tumor outgrowth under hormone-depleted conditions. In accordance, *KMT2C* is one of the most frequently mutated genes in ER-positive breast cancer with *KMT2C* deletion correlating with significantly shorter progression-free survival on anti-estrogen therapy. From a therapeutic standpoint, KMT2C-depleted cells that develop hormone-independence retain their dependence on ERα, displaying ongoing sensitivity to ERα antagonists. We conclude that KMT2C is a key regulator of ERα activity whose loss uncouples breast cancer proliferation from hormone abundance.

## Introduction

Cancer-specific transcriptional programs are foundational to the development of oncogenic phenotypes. For example, oncogenes such as MYC and BRAF drive unique gene expression signatures that have been shown to be essential for transformation and cancer maintenance [1–5]. From a therapeutic vantage, reversal of these transcriptional programs is critical to the efficacy of most forms of targeted therapy.

Recent large-scale genomic analyses have identified key chromatin modifications permissive of such tissue-specific and cancer-specific transcriptional programs [6, 7]. Among the most significant of these chromatin modifications is methylation at histone H3 lysine 4 (H3K4me), generally marking regions of active and poised transcription. H3K4 histone methyltransferases mono-methylate H3K4, di-methylate H3K4, or tri-methylate H3K4 via their enzymatically active SET domain. Trimethylation (H3K4me3) of these residues is observed to be more abundant at promoter regions while monomethylation (H3K4me1) is more abundant at enhancer regions [8].

Interestingly, data from several large-scale cancer sequencing studies have identified *KMT2C* (also referred to as *MLL3*), which encodes an H3K4 histone methyltransferase, as one of the most commonly mutated genes in breast cancer with a mutation frequency of approximately 8% [9–11]. KMT2C, which is partially redundant with its most closely related homolog, KMT2D, is thought to be an H3K4me1/2 methyltransferase active predominantly at enhancer regions of the genome [12, 13]. The high prevalence of *KMT2C* mutation suggests that KMT2C may have important functions in breast cancer, which is so often characterized by its dependence upon the transcription factor, ERα. Here, we investigated the role of KMT2C in breast cancer pathogenesis and found it to be an essential ERα coactivator.

## Results

### KMT2C is one of the most frequently mutated genes in breast cancer

**Fig. 1:**
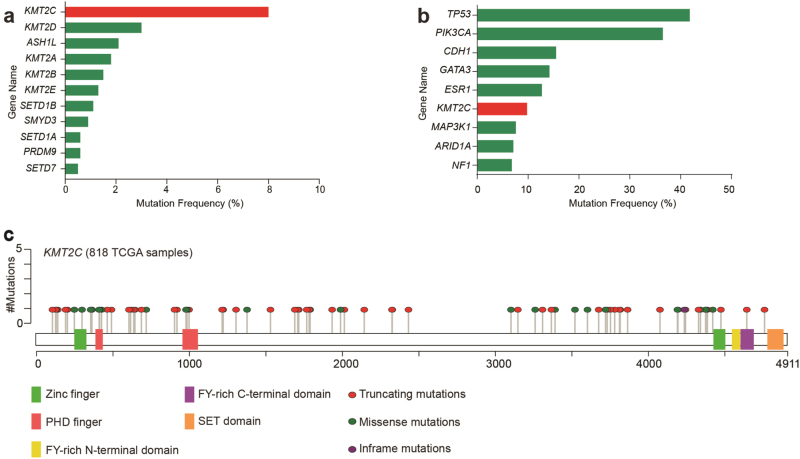
KMT2C is the most frequently mutated H3K4 histone methyltransferase in breast cancer. **a** Mutation frequencies of H3K4 histone methyltransferases in breast cancer tissue samples from the TCGA data set [10] (*n* = 818). **b** Percentage of cases with gene mutations detected in the MSKCC-IMPACT metastatic breast cancer patient cohort [60] (*n* = 746). **c** Diagram of KMT2C domains with the locations of the identified TCGA mutations. *y*-axis corresponds to the number of cases with indicated mutation (*n* = 818)

Within the H3K4 methyltransferase family, *KMT2C* is by far the most commonly mutated member with a frequency of approximately 8% in TCGA breast cancer samples [10] (Fig. 1a). To assess the presence and persistence of *KMT2C* mutation in breast cancer pathogenesis, we examined *KMT2C* mutation frequency in metastatic tumors. *KMT2C* mutations were found in 9.8% of over 700 metastatic breast tumors analyzed, placing it among the most commonly mutated genes in breast cancer (Fig. 1b). The majority of *KMT2C* mutations are frameshift, truncation or missense mutations, with a substantial proportion predicted to interfere with expression of the carboxy-terminal SET domain (Fig. 1c). The mutations appear to occur in all breast cancer subtypes with a relatively even distribution (Fig S1). Together this data suggests that KMT2C may act as a breast cancer tumor suppressor and might be a candidate regulator of H3K4me in these tumors.

### KMTC2 knockdown reduces cell proliferation of ER+ breast cancer cells

In order to test the function of KMT2C in breast cancer, we cloned two short hairpins against *KMT2C* [14] (shKMT2C). We found that both short hairpins, shKMT2C#1 and shKMT2C#2, reduce expression of KMT2C by 60–70% without affecting expression of its most closely related homologs, *KMT2A*, *B*, or *D* (Fig. S2A). We stably expressed shKMT2C#1 and shKMT2C#2 in a panel of cell lines representative of the clinical subtypes of breast cancer and found similar degrees of knockdown across the panel (Fig. S2B). All cell lines used underwent next-generation sequencing and showed no clear deleterious mutation in *KMT2C* (Supplementary Table 1). To confirm sufficient knockdown of KMT2C protein levels, we used MCF7 cells that have been engineered to express HA at the C-terminal end of an endogenous *KMT2C* allele (KMT2C-HA cells). Expression of shKMT2C #1 and shKMT2C #2 in these cells resulted in knockdown of KMT2C-HA by immunoblotting (Fig. S2C), while not affecting protein levels of KMT2A, B or D (Fig. S2D).

**Fig. 2:**
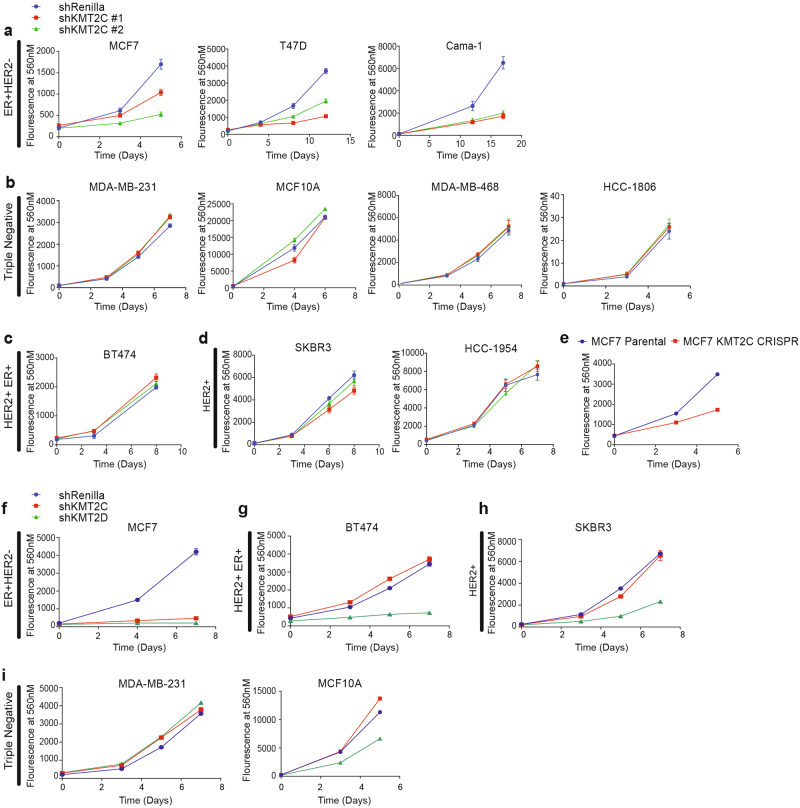
KMT2C loss inhibits proliferation of ER+ cells. **a**–**d** Breast cancer cells stably expressing either shRenilla or shKMT2C were assayed for proliferation using the alamarBlue cell viability assay. Values correspond to the mean of six experimental replicates ± s.e.m. All cells are cultured in full serum containing media **e**. Parental and MCF7 KMT2C CRISPR cells were assayed for proliferation using the alamarBlue cell viability assay. Values correspond to the mean of six experimental replicates ± s.e.m. Data correspond to one representative assay from a total of two or three independent assays. **f**–**i** Indicated breast cancer cells stably expressing either shRenilla, shKMT2C or shKMT2D were assayed for proliferation using the alamarBlue cell viability assay. Values correspond to the mean of six experimental replicates ± s.e.m.

We subsequently used the shKMT2C-expressing breast cancer models to assay the effects of KMT2C loss on cell proliferation. KMT2C knockdown resulted in a 40–70% reduction in proliferation selectively in the three ER+HER2− cell lines examined, MCF7, T47D and Cama-1 (Fig. 2a). In contrast to the effects seen in the ER+HER2− cells lines, KMT2C knockdown had no effect on the proliferation of the ER+HER2+ cell line BT474, the ER-HER2+ cell lines, SKBR3 and HCC1954 and the triple negative cell lines, MDA-MB-231, MDA-MB-468, MCF10A and HCC1806 (Fig. 1b–d). Similarly, CRISPR/Cas9nickase mediated knockout of *KMT2C* suppressed proliferation in MCF7 cells (Fig. S3, Fig. 1e) but not in MCF10A cells (data not shown). Given the close homology between *KMT2C* and *KMT2D*, we examined whether loss of KMT2D would have similar effects on the proliferation of ER+ breast cancer cell lines. We expressed shKMT2D in a representative panel of breast cancer cell lines resulting in approximately 50–70% knockdown of *KMT2D* mRNA (Fig. S4). We observed that while the effects of KMT2C loss on proliferation were specific to the ER+HER2− cell lines, loss of KMT2D suppressed the proliferation of all cell lines tested except MDA-MB-231 (Fig. 2f–i). This suggested that the effects of KMT2C on ER+/HER2− breast cancer proliferation may be unique and not shared by all H3K4 methyltransferases, prompting us to further investigate its role in estrogen response.

### KMT2C loss suppresses ERα target gene expression

Given the effects of KMT2C loss on estrogen-driven breast cancer models, we next examined whether and how KMT2C might be regulating signaling by ERα. We assessed the effect of KMT2C loss on global receptor activity by evaluating ERα expression, phosphorylation and conformational stability. We found no major changes in the levels of total ERα in both MCF7 and T47D cells (Fig. S5A and S6). In addition, we found no changes in phosphorylation of ERα (S118 or S104/S106) or in the HSP90 dependence of ERα after KMT2C knockdown (Fig. S5 and S6). Given the lack of effects upon ERα expression or conformation, we next sought to determine whether there might be changes in aspects of ERα-regulated gene expression.

**Fig. 3:**
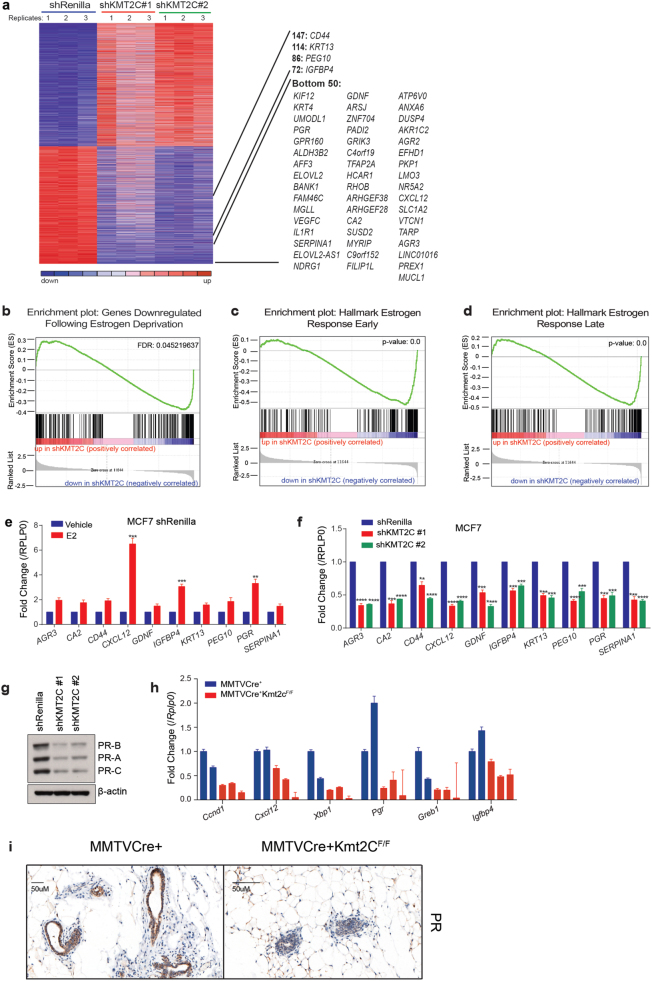
KMT2C loss suppresses ERα target gene expression. **a** Supervised analysis of the 7938 differentially expressed genes between shRenilla and shKMT2C (shKMT2C#1 and #2 combined) MCF7 cells. All cells are cultured in full serum containing media. **b** GSEA showing 3857 genes downregulated in shKMT2C cells are enriched among genes downregulated following 5-day estrogen deprivation of shRenilla cells (≥3-fold). **c** GSEA of 3857 genes downregulated in shKMT2C as compared to the Hallmark Estrogen Response Early Geneset (Broad Institute). **d** GSEA of 3857 genes downregulated in shKMT2C as compared the Hallmark Estrogen Response Late Geneset. **e** mRNA levels; values correspond to the mean of three replicates ± s.e.m. E2, estradiol. **f** mRNA levels; values correspond to the mean of three replicates ± s.e.m.; two-tailed Student’s *t*-test with a desired FDR = 1% was used to determine statistical significance; **P < 0.01, ***P < 0.001, ****P < 0.0001. Data correspond to one representative assay from a total of two or three independent assays. **g** Immunoblot; β-actin used as loading control. **h** mRNA levels, as measured by qRT-PCR, from RNA taken from the mammary glands of 12-week old virgin females. Values correspond to the mean of three replicates ± s.e.m. **i** Representative slides of PR expression in mammary glands from 12-week-old virgin females

To this end, we performed RNA sequencing on control shRenilla and shKMT2C MCF7 cell lines (Fig. 3a). We then compared the gene expression changes following KMT2C knockdown to those following 5-day hormone deprivation of control shRenilla cells. We found that genes that were downregulated following KMT2C knockdown were strongly enriched among genes downregulated following hormone deprivation (Fig. 3b). Additionally, upon comparing the downregulated genes in the KMT2C knockdown cells to Hallmark gene sets, we found strong enrichment specifically among ERα target gene sets (Fig. 3c, d). In further support, we performed RNA sequencing of ER+T47D cells following KMT2C knockdown. We again found genes downregulated by KMT2C loss to be highly enriched among ERα target gene sets (Fig. S7A–C). We validated the differential gene expression data using qRT-PCR on a panel of ERα target genes whose expression was stimulated with E2 (Fig. 3e). Expression of all of these transcripts was reduced by 5–60% following KMT2C knockdown in MCF7 T47D and Cama-1 cells (Fig. 3f, S7D, E). To further confirm these findings, we examined protein expression of progesterone receptor (PR), a well-established ERα target, and found it to be decreased by approximately 70% following KMT2C loss (Fig. 3g).

To determine whether Kmt2c might regulate ERα activity in another context where ligand-activated ERα regulates cellular proliferation and differentiation, we examined the role of KMT2C in the murine mammary gland. We generated mice with floxed alleles of *Kmt2c* (Fig. S8A-C) and crossed them with mice expressing Cre recombinase under the control of the mammary-specific MMTV promoter (Fig. S8D-F). We assayed for several ERα target genes in both control and Kmt2c knockout glands. We found reduced expression of multiple ERα target genes in the Kmt2c knockout mice to levels that were approximately 40–90% below levels seen in control mice (Fig. 3h). In addition, we assayed for nuclear expression of the PR, a known ERα-target gene, by IHC and found significant reductions (Fig. 3i). These data confirm that KMT2C regulates key aspects of the estrogen response program while not globally controlling ERα expression or activity.

### KMT2C loss suppresses ERα function at enhancer sites

**Fig. 4:**
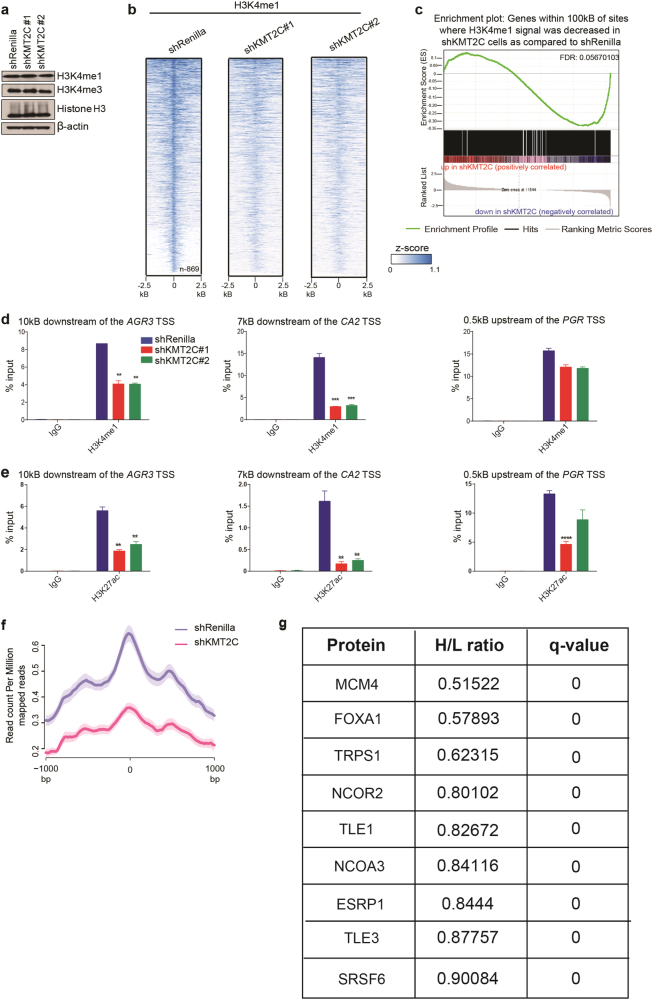
KMT2C loss results in site-specific loss of H3K4me1 and H3K27ac at ERα enhancers. **a** Immunoblot; β-actin used for loading control. **b** Normalized heatmaps for H3K4me1 occupancy in shRenilla, shKMT2C#1 and #2 cells among the 869 differential sites. Heatmaps centered at the peak summit. All cells are cultured in full serum containing media **c** GSEA of 3857 genes significantly downregulated in MCF7 shKMT2C cells (*p*-val < 0.05) as compared to ranked genes with a ≥ 25% reduction in H3K4me1 at enhancers in shKMT2C cells. **d** qChIP analysis; IgG used as negative control. Values correspond to mean percentage of input ± s.e.m. of triplicate qPCR reactions of a single replicate. **e** qChIP analysis; Two-tailed Student’s *t*-test with a desired FDR = 1% was used to determine statistical significance; **P < 0.01, ***P < 0.001, ****P < 0.0001. Data correspond to one representative assay from a total of three independent assays. **f** Average H3K27ac binding in shRenilla and shKMT2C MCF7 cells at sites of H3K4me1 loss centered at peaks ± 1kB. shKMT2C#1 and #2 H3K27ac peaks were averaged to generate the shKMT2C profile. **g** List of differentially bound ERα binding partners in shKMT2C#2 vs. shRenilla MCF7 cells. H/L refers to the ratio of peptides found in the heavy (shKMT2C) vs the light (shRenilla) labeled lysates. List was restricted to previously established ERα binding partners [23]. Assay done in triplicate. Full list found in Supplementary Table 2

It has been speculated that H3K4me may help demarcate regions for ERα binding and activity [15]. Given the known enzymatic activity of KMT2C in generating H3K4me in other cell types [16], we hypothesized that KMT2C might regulate ERα activity by placing these marks at ERα binding sites. We first examined total H3K4me1 and H3K4me3 levels by immunoblotting and found no change following KMT2C loss, implying that KMT2C is not the sole methyltransferase responsible for these marks in ER+ cells (Fig. 4a). We next examined the pattern of genome-wide H3K4me1 and H3K4me3 following KMT2C loss using ChIP-sequencing. Whereas KMT2C loss had no meaningful effect upon H3K4me3 across the genome (data not shown), it appeared to selectively alter H3K4me1 at 869 affected loci, or 1% of the total 84,484 sites of H3K4me1 occupancy in control cells (Fig. 4b, Fig. S9).

To analyze the functional significance of these sites of H3K4me1 loss, we integrated these data with the RNA sequencing data (Fig. 3a) and found a strong enrichment for sites with H3K4me1 loss with sites whose nearby genes showed diminished expression with KMT2C knockdown (Fig. 4c). To be sure, we also included assessment of decreased H3K4me1 at promoter regions and found that it also correlated with decreased gene expression (Fig. S10). However, the 73 promoter sites that correlated with reduced gene expression accounted for a small portion of overall KMT2C-mediated H3K4me1 loss. This is again consistent with previous studies indicating that H3K4me1 demarcates activity at enhancers as opposed to promoters. To confirm these effects upon ERα enhancers, we examined H3K4me1 at known ERα enhancer sites and found decreases in H3K4me1 from 15% at the *PGR* enhancer to 80% at the *CA2* enhancer (Fig. 4d), given the prior data that ERα regulates gene expression through its actions at both promoter and enhancer sites, our integrated H3K4 methylation and RNA sequencing data points to KMT2C predominantly altering ERα activity via its effects upon ERα enhancer sites.

As the H3K4me1 mark is known to demarcate both regions of active transcription and regions “poised” for transcription, histone H3 K27 acetylation (H3K27ac) can be used to more strictly delineate those sites that are actively engaged in transcription [17]. To determine if KMT2C was regulating active enhancers, we assayed for H3K27ac at the *AGR3*, *CA2*, and *PGR* enhancer sites, where we saw loss of H3K4me1. We found significant reduction of H3K27ac at these sites (Fig. 4e). We went on to perform H3K27ac ChIP-sequencing on KMT2C knockdown MCF7 cells and found that H3K27ac was coordinately reduced at sites of H3K4me1 loss (Fig. 4f, Fig. S11). These data further establish a model in which KMT2C is responsible for depositing H3K4me1 selectively at active ERα enhancers thereby facilitating a key aspect of the ERα regulon.

H3K4me1 may facilitate enhancer function by promoting binding of any of a number of components necessary for site-specific transcription. It has been speculated that DNA-binding of ERα itself may require H3K4 methylation and/or pioneer factor binding [15, 18, 19]. To test this hypothesis, we examined ERα ChIP-sequencing in KMT2C knockdown cells. Unlike the case with H3K4me1/H3K27ac, we did not observe major loss of ERα binding at sites of changes in gene expression (Fig. S12A). Not surprisingly, we did find a handful of sites with decreased ERα binding and concomitant decreases in proximal gene expression (Fig. S12B). However, the 12 genes accounted for by loss of ERα binding were only 0.3% of the total gene expression changes observed. Moreover, we observed no change in levels of ERα at > 75% of ERα binding sites proximal to sites we found to have KMT2C dependent H3K4me1 (Fig. S12A). As such, the data points to KMT2C regulating ERα enhancer function perhaps via its facilitation of essential ERα coactivators, but not via recruitment of ERα itself.

In order to nominate potential coactivators involved in mediating the effects of KMT2C upon on ERα, we performed de novo motif analysis upon a 1kB radius around sites where H3K4me1 had decreased in shKMT2C cells. We found that these regions were significantly enriched over randomly selected background sequences for the well-known ERα pioneer factor, FOXA1 [15] as well as the ERα coactivator, AP-1 [20] (Fig. S13). While a number of motifs were considered to be significant in this analysis, it is notable that these two are both known factors necessary for ERα action. To further screen for potential intermediaries between KMT2C and ERα activity, we looked for changes in the ERα interactome. Given the large number of potential interactions, we sought to restrict this search to interactions occurring on DNA. To this end, we employed rapid immunoprecipitation mass spectrometry of endogenous proteins (RIME) [21, 22] with shKMT2C#2 MCF7 cells cultured in heavy SILAC media and shRenilla MCF7 cells cultured in light SILAC media. We compared our list of peptides differentially bound to ERα with previously published ERα-binding partners [23]. These data revealed only modest changes with most interactions comparable between cells (Fig. 4h). However, they did reveal reduced binding of ERα to MCM4, FOXA1, and TRPS1 by over 1/3 in shKMT2C cells and no proteins showing enhanced binding (Fig. 4h, full list Supplemental Table 2). Taken together, the findings point to a possible role for KMT2C in regulation of the interaction between ERα and FOXA1, a well-established pioneer factor essential for ERα activity.

### KMT2C loss promotes hormone-independent ER+ breast cancer cell proliferation

**Fig. 5:**
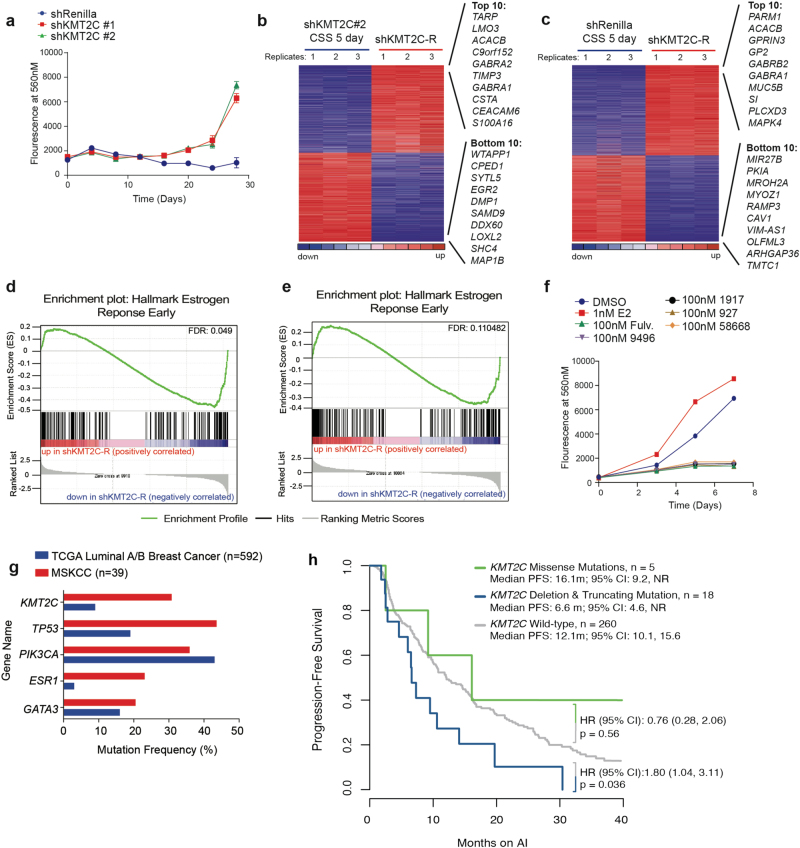
KMT2C loss promotes hormone independent outgrowth. **a** Cells were assayed for proliferation in CSS media using the alamarBlue viability assay. Values correspond to the mean of six experimental replicates ± s.e.m. Data correspond to one representative assay from a total of two independent assays. **b** Supervised analysis of the 8622 differentially expressed genes between shKMT2C#2 cells in CSS media for 5 days and shKMT2C-R. CSS, charcoal stripped media. **c** Supervised analysis of the 9398 differentially expressed genes between shRenilla cells in CSS media for 5 days and shKMT2C-R. **d** GSEA of 4347 genes significantly downregulated in **b** as compared to the Hallmark Estrogen Response Early Geneset. **e** GSEA of 4559 genes significantly downregulated in **c** as compared to the Hallmark Estrogen Response Early Geneset. **f** MCF7 shKMT2C-R cells were treated with DMSO, estradiol (E2), fulvestrant (Fulv.), AZD9496 (9496), ARN1917 (1917), GDC927 (927), or RU58668 (58668) at indicated doses. Proliferation assayed using the alamarBlue viability assay. Values correspond to mean of 6 experimental replicates ± s.e.m. **g** Percent of cases with mutations detected in patient samples (MSKCC [50]) compared to those in TCGA Luminal A/B [10]. **h** Progression free survival for ER+ breast cancer patients on single agent aromatase inhibitor (AI)

Given the necessity for KMT2C in estrogen-driven growth, we hypothesized that loss of KMT2C might facilitate a hormone-independent growth state as might occur in the context of hormone deprivation therapy. To assess the role of KMT2C in this context, we took advantage of the ability of MCF7 cells to grow in hormone-deprived conditions after 3–4 months [24, 25]. Using similar conditions, we observed that KMT2C-deficient MCF7 cells displayed a much more rapid time to growth with cells emerging in as little as 2 weeks (Fig. 5a). Consistent with these differences, control shRenilla cells continued to be growth arrested for 10–12 weeks beyond the time KMT2C knockdown cells grew. By contrast, we found that this rapid outgrowth was not observed in KMT2D knockdown cells (Fig. S14).

To investigate the basis of hormone-independent growth in this context, we established a hormone-independent MCF7 shKMT2C#2 cell line referred to as shKMT2C-R. Next-generation DNA sequencing (IMPACT) of the shKMT2C-R cells revealed no obvious acquired mutations compared to parental cells that could explain the rapid outgrowth in hormone-deprived media (Supplemental Table 3).

We assayed for KMT2C mRNA levels in these resistant cells and found that KMT2C levels remained suppressed (Fig. S15A, B). We also performed H3K4me1 ChIP-sequencing in these cells and found that the original 869 sites with reduced H3K4me1 continued to have reduced levels of H3K4me1 (Fig. S15C, D). These data suggested that the development of hormone-independent growth was not merely through replacement of KMT2C activity upon ERα enhancers by upregulating a different H3K4 methyltransferase (or suppressing the demethylase).

To interrogate whether there might be any unique dependencies of these cells, we performed RNA sequencing on the shKMT2C-R cells and compared their gene expression levels to that of parental shKMT2C#2 or shRenilla MCF7 cells maintained in hormone-deprived media for 5 days (Fig. 5b, c). We found that genes downregulated in shKMT2C-R cells were enriched for ERα target genes (Fig. 5d, e) and genes implicated in responses to growth factor stimulation (Fig S16). We also examined genes upregulated in the shKMT2C-R cells and found enrichment for genes involved in metabolic pathways including fatty acid, inositol phosphate, glucose and amino acid metabolic pathways. However, no individual pathway appeared significant when we corrected for multiple hypothesis testing (data not shown).

As the gene expression data suggested an ongoing suppression of ERα target genes, we examined whether these cells had become insensitive to the action of estrogen. We tested the response of shKMT2C-R to estradiol (E2) and found that the shKMT2C-R cells continued to respond to E2 by increasing their proliferative rate (Fig. 5f). These data suggest an ongoing responsiveness to the estrogen stimulated growth program, but a capability to proliferate in the absence of ERα ligands. To determine if unliganded ERα was still promoting growth in these cells, we examined their response to ERα antagonism. Similar to E2 response, shKMT2C-R cells were still dependent on ERα as they were sensitive to inhibition by multiple selective ERα degraders including fulvestrant, AZD9496, ARN1917, SRN927, and RU58668 (Fig. 5f). These data together suggested that the shKMT2C-R cells continue to be responsive to an E2-driven growth program while unliganded ERα maintains essential roles under hormone-deprived conditions.

Given the dual effects of KMT2C in promoting hormone-dependent tumor growth while suppressing hormone-independent tumor growth, we examined the clinical outcomes of patients with KMT2C loss. First, we applied the gene expression signatures derived from our shKMT2C cells (Fig. 3a) and our shKMT2C-R cells (Fig. 5c, d) and compared them to gene expression data from 1209 Luminal A and B breast cancer patients in the METABRIC data set [26]. We were able to separate the patients into three groups (high, mid, low) based on the similarity of their gene expression data to the derived gene expression signatures (Fig. S17) Those with a high score had a gene expression profile most similar to that of KMT2C knockdown. We found that patients with high scores were more likely to be have poorer overall survival and be Luminal B in subtype than Luminal A (Fig. S17).

Together these data suggest that whereas KMT2C loss suppresses estrogen-driven tumor growth, it can also facilitate emergence of growth in hormone-depleted conditions. In keeping, we noted that *KMT2C* mutation prevalence was increased to over 30% among patients with metastatic, hormone-refractory breast cancer compared to the ~9% mutation frequency observed in primary, untreated Luminal A/B TCGA breast cancer (Fig. 5g, S18). In support, we went on to look at patients with metastatic breast cancer who received single agent aromatase inhibitor and compared patients wildtype or mutant for *KMT2C* (truncation mutations and deletions). We found that these patients with unambiguously pathogenic *KMT2C* mutations had significantly shorter progression-free survival (PFS) on hormone deprivation therapy than patients with wildtype *KMT2C*, further supporting a role for KMT2C in hormone resistance (Fig. 5h).

### ERα is reprogrammed in the shKMT2C-R cells

**Fig. 6:**
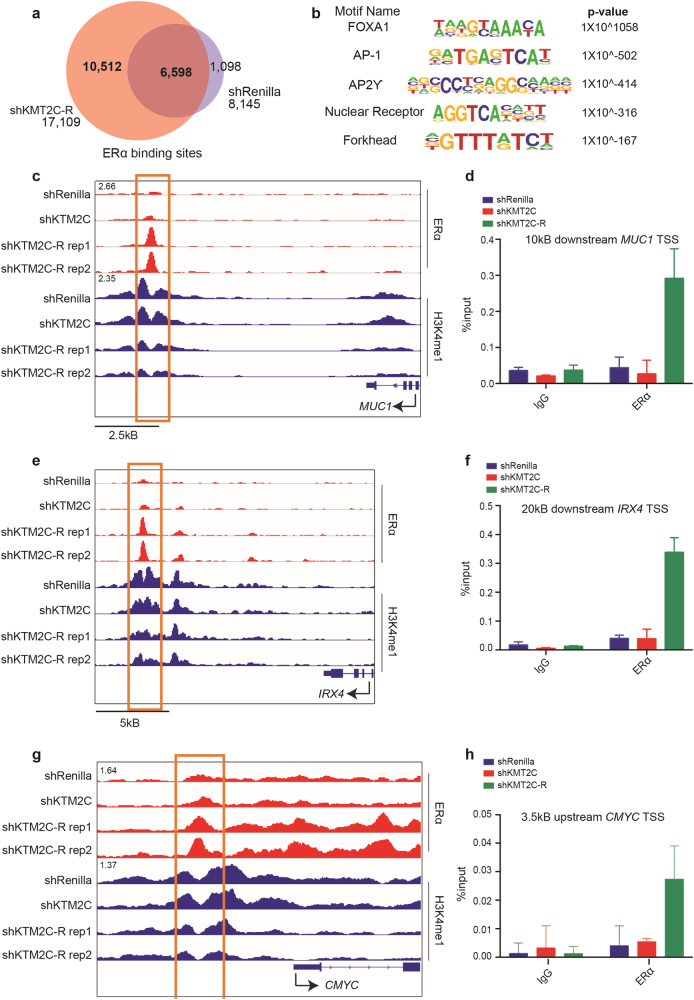
ERα is reprogrammed in shKMT2C-R cells. **a** Venn diagram showing overlap between shKMT2C-R (in CSS, red) and shRenilla (in full serum, purple) ERα binding sites. **b** HOMER motif analysis at the 10,512 novel ERα loci in shKMT2C-R. Full list in Fig. S17. **c**, **e**, **g** IGV browser views for ERα in shRenilla and shKMT2C-R cells. Sites of increased ERα binding outlined in orange. For *CMYC*, orange outline encompasses a previously defined AP-1 dependent ERα site [34]. **d**, **f**, **h** qChIP analysis. IgG used as negative control. Values correspond to mean percentage of input ± s.e.m. of triplicate qPCR reactions of a single replicate. Data correspond to one representative assay from a total of two independent assays

Our findings for shKMT2C-R cells demonstrated that they no longer required estrogen to support growth but maintained a dependence of ERα expression and reactivated ERα gene expression programs. To examine the site of action of ERα in this context, we performed ERα ChIP-sequencing of these cells. Whereas we continued to identify many sites seen in the shRenilla cells (*n* = 6598), we also identified over 10,000 unique ERα binding sites (Fig. 6a). We performed motif analysis on these distinct sites and found motifs of FOXA1 and AP-1,to be enriched (Fig. 6b, S19). AP-1, in particular, has been previously shown to play a major role in the hormone-independent outgrowth of ER+ breast cancer [27–33]. To test the relevance of AP-1 in shKMT2C-R cells, we looked for ERα binding nearby to target genes, *IRX4* and *MUC1*, whose expression is known to be regulated by AP-1 dependent ERα activity [20]. We also assayed ERα binding at a known AP-1 dependent ERα binding site upstream of *c-MYC*, a key driver of hormone-independent growth [34]. We found significant increases in ERα binding downstream of *IRX4* and *MUC1* and at the known AP-1-dependent ERα binding site in shKMT2C-R cells as compared to control cells (Fig. 6c–h). These data raise the possibility that AP-1 may, in part, be responsible for reprogramming ERα under conditions of KMT2C loss.

## Discussion

In this report, we utilize tumor genome sequencing analyses to help uncover the H3K4 methyltransferase KMT2C as a critical regulator of hormone-dependent ERα activity. Although it is unlikely that KMT2C will prove to be the only histone modifier that regulates ERα function, the findings in this report suggest it to be a key regulator of the estrogen responsive phenotype with major implications for hormone therapy of breast cancer.

The initial suggestion for a role for KMT2C in estrogen dependence comes from its frequent mutation in primary breast cancer. As most mutations appear to be deleterious, we modeled KMT2C loss in breast cancer cell lines. We were surprised to find that KMT2C loss led to a selective proliferative defect in ER+-driven breast cancers. These data were remarkable as no breast cancer model we tested showed an obvious growth advantage from loss of KMT2C. Given previous observations about the interaction between H3K4me and nuclear receptor activities [16, 35–45], we speculated that KMT2C might be an essential regulator of ERα.

To examine the mechanisms of KMT2C-mediated regulation of ER+ breast cancer proliferation, we analyzed the effect of its loss upon established gene expression signatures of ligand-activated ERα. KMT2C loss downregulated estrogen responsive gene expression. These effects of KMT2C loss are notable in light of the previously reported effects of KMT2C loss in other contexts, including hematopoietic cells and stem cells, where its loss is predominantly associated with an increase in stem-like properties and chemotherapy resistance [20, 46, 47]. Specifically, previous work studying KMT2C in mouse mammary stem cells suggested it to be tumor-promoting [47], while our own work on human ER+ breast cancer cells suggest KMT2C to be either tumor-suppressive or tumor-promoting, depending on hormone availability. In support of KMT2C acting as a tumor suppressor, KMT2C loss has been shown to contribute to urothelial carcinomas by forming a tumor suppressive complex with p53 [45]. This disparity in KMT2C function across multiple tissue types points to the importance of cellular context in understanding the functions of KMT2C. Whereas this gene is among the most frequently mutated genes in all of cancer [5], the dominant effects of its loss appear to be cell type specific. Whether there are key shared functions of KMT2C across different tissue types or malignancies remains to be seen.

Early analyses of KMT2C biochemical activities and KMT2C knockout cells pointed to an essential role for this protein in generating the H3K4me1 mark associated more frequently with enhancer sites [16]. In accordance, we found H3K4me1, but not H3K4me3, to be lost specifically at ERα enhancer sites in KMT2C knockdown cells. Moreover, ChIP-sequencing of H3K27ac demonstrated that H3K4me1 loss coincided with sites of H3K27ac loss, implying that these were sites of active ERα driven transcription. These data specify the mechanism of KMT2C regulation of ERα function—through enabling transcriptional activity at ERα enhancer sites.

To understand the basis for how H3K4me1 might influence ERα activity, we conducted ERα ChIP-sequencing and found no significant loss of ERα binding despite the loss of expression of proximal ERα target genes. How then does H3K4me1 influence ERα activity at these enhancer sites? We note that in examining the region surrounding these bindings sites, it appears that sites predicted for ERα binding partners, such as FOXA1 and AP-1, are prevalent. In addition, we observed that KMT2C loss led to decreases in ERα interactions with multiple known binding factors including MCM4, FOXA1 and TRPS1. Finally, as independent proof of potential interactions of KMT2C and FOXA1, Jozwik et al. recently uncovered a direct interaction between KMT2C and FOXA1. How FOXA1, AP-1, and other specific factors ultimately coordinate KMT2C-dependent ERα activity will be an area for further investigation.

Finally, we sought to understand the basis for tumors selecting for loss of KMT2C. We note that not all *KMT2C* mutations seen in breast cancer will recapitulate loss of function but given that over 55% of *KMT2C* mutations are truncation mutations and given the lack of a specific hotspot mutation, our KMT2C knockdown cells provided a key model to study the functional role of KMT2C in breast cancer. We queried *KMT2C* mutation frequency at different stages of breast cancer progression and observed a ~7% mutation rate in primary breast cancer, a similar rate (~10%) in metastatic breast cancer and a higher rate in hormone-independent breast cancer (~30%). These data were in some contrast to the proliferative defects we observed in ER+ cell lines growing in hormone-containing media. We investigated whether there might be a selective advantage for its loss in estrogen-depleted conditions. Here, we indeed observed that KMT2C loss led to markedly accelerated outgrowth of cells. These data were further corroborated by examining how loss of KMT2C impacted patient survival. Among these patients, KMT2C loss was correlated with poorer survival outcomes and shortened PFS on aromatase inhibitor therapy. In accordance, it was recently published that low KMT2C expression was significantly associated with poor outcome [48]. Together, these data suggest that KMT2C loss is likely to have complex effects on the clinical behaviors of breast cancers. We anticipate that under some circumstances the loss of KMT2C will slow estrogen-driven tumor growth and potentially be associated with favorable outcomes. In other contexts, loss of KMT2C may be permissive for tumors developing an estrogen-independent growth program, perhaps only in selected genotypes where another growth program is sufficiently active. Moreover, even in this context, our data would suggest ERα may still be targetable as our shKMT2C-R cells were still sensitive to SERD-mediated ERα antagonism. Indeed, our analyses of these shKMT2C-R cells suggest that KMT2C loss was permissive for large-scale reprogramming of ERα such that it acquired thousands of new binding loci. The function of these new binding sites is not yet known, but the sensitivity of the cells to ERα inhibitors suggest there may be key oncogenic functions gained. It will thus be imperative to further understand the growth requirements for different KMT2C-mutated tumors to better utilize this biomarker in selecting the types of endocrine therapy most appropriate for this common clinical entity.

## Methods

### Plasmids

SGEP (pRRL) was a kind gift from C. Sawyers. psPAX2 and pVSVG vectors were kind gifts from P. Chi.

### Cell Lines

All cell lines were maintained at 37 °C and 5% CO_2_ in a humidified atmosphere. SKBR3, BT-474, Cama-1, T47D, MCF10A HCC1954 and MDA-MB-231 MDA-MB-468 and HCC1806 cell lines were obtained from the American Type Culture Collection, and MCF7 Tet-On cells were obtained from Clontech. HEK293CT cells were a kind gift from P. Chi. SK-BR-3, Cama-1, HCC1954 and MDA-MB-468 and MCF7 cells were grown in DMEM-F12, T47D and HCC1806 and MDA-MB-231 cells were grown in RPMI, and HEK293CT cells were grown in DMEM, supplemented with 10% heat-inactivated FBS, 100 µg/ml penicillin, 100 mg/ml streptomycin and 4 mM glutamine. MCF10A cells were grown in DMEM-F12 supplemented with 5% heat-inactivated horse serum, 100 µg/mL penicillin, 100 mg/mL streptomycin, 4 mM glutamine, 20ng/mL EGF, 500 µg/mL Hydrocortisone, 100ng/mL Cholera Toxin, and 10 µg/mL Insulin. All cell lines tested negative for mycoplasma. No cell line used is in the database of commonly misidentified cell lines. MCF-7, SKBR3 and MDA-MB-231 were authenticated by STR profiling. Cama-1, and T47D and MCF10A, MDA-MB-468, HCC-1806, HCC-1954 were authenticated by next-generation (IMPACT) sequencing.

### Clinical samples (MSKCC set)

We previously described use of a 410-gene targeted capture-based sequencing platform (MSK-IMPACT [49]) to analyze 39 tumors with either recurrence of disease after receiving adjuvant therapy or World Health Organization-defined progression of metastatic disease on therapy for genomic alterations [50]. Informed consent was received from all patients.

### Sequencing breast cancer cell lines for the presence of KMT2C alterations

MCF7, T47D, Cama-1, SKBR3, BT-474, MCF10A, MDA-MB-468, HCC-1954, HCC-1806 and MDA-MB-231 cells were sequenced using the next-generation sequencing (IMPACT) assay as previously described [49].

### Generation of MCF7 KMT2C-HA by CRISPR/Cas9

The following guide RNA sequence targeting KMT2C exon 60 was selected using the Optimized CRISPR Design tool (http://crispr.mit.edu [51]): AGCCGCCCGCTGAGCTAGCA.

DNA oligonucleotides were purchased from IDT and cloned into px458-GFP vector [52]. For homologous recombination, we purchased a custom IDT Ultramer 200 bp repair template with the HA sequence (TACCCATACGATGTTCCAGATTACGCT) directly upstream the KMT2C stop codon. This 200 bp fragment contained two silent mutations, one to remove the PAM site (TCC > TTC) and another to introduce a HincII restriction enzyme (GTGAAC > GTTAAC) site upstream of the HA tag. The 200 bp fragment also contained 87 bps of homology to KMT2C exon 60 upstream of the HA tag and 83 bp of homology to the KMT2C 3′UTR downstream of the stop codon. 12ug of the targeting construct and 240 nM repair template were nucleofected into MCF7 cells using the Lonza Nucleofector V kit and Program P020 on the Nucleofector device. Nucleofected cells were single cell sorted based on GFP positivity 48 h following nucleofection. Clones were screened for the presence of successful HA insertion by *KMT2C* exon 60 PCR and subsequent restriction enzyme digest with HincII. Positive clones show two digested products while negative clones only had a single undigested band. A single positive clone, called KMT2C-HA, containing the HA coding sequencing downstream KMT2C was selected to carry out further studies. As a control, we used one of the negative clones from the screening process, called KMT2C crispr control.

### Lentiviral Infections

We plated HEK293CT packaging cells at 2.5 × 10^7^ cells/ tissue culture dish (10 cm in diameter) and transfected them with 2.25 µg of the SGEP lentiviral vector (encoding shRenilla, shKMT2C #1 or shKMT2C #2), 2.25 µg of psPAX2 and 0.5 µg of pVSVG with X-tremeGENE HP(Roche) according to the manufacturer’s protocol. Conditioned medium containing recombinant lentivirus was collected and filtered through non-pyrogenic filters with a pore size of 0.45 µm (Millipore). Samples of these supernatants were applied immediately to target cells, which had been plated 18 h before infection at a density of 3 × 10^6^ cells/tissue culture dish (10 cm in diameter). Polybrene (Sigma) was added to a final concentration of 8 µg/ml, and supernatants were incubated with cells for 12 h. After infection, cells were placed in fresh growth medium and cultured as usual. Selection with 5–7 µg/ml puromycin was initiated 48 h after infection.

### Proliferation assays

About 500–1000 cells in 200 µL of media were seeded per well of a 96-well plate with six replicates per sample. If necessary, cells were treated the following day, (Day 0). On days when the plates were measured, 25 µL of Resazurin (R&D Systems AR002) was added per well followed by 4 h incubation at 37 °C. Plates were then read using a SpectraMax M5 (Molecular Devices) and results were analyzed with Softmax Pro 6.2.2 software with an Endpoint Readtype (Excitation: 560 nM, Emission: 590 nM). Results were normalized to blank media with no cells.

For long-term estrogen deprived proliferation assay (Fig. 2f), 5000 cells were seeded in 200 µL of charcoal stripped media. Media was replenished twice a week during the course of the proliferation assay.

### Immunoblotting

Cells were washed once with cold PBS and scraped off the plate with a rubber policeman. The cell suspension was briefly centrifuged to pellet cells, PBS was removed, and the cell pellet was stored at −80 °C until lysis. For cell lysis, pellets were resuspended in a non-denaturing lysis buffer (Cell Signaling Technology) supplemented with protease and phosphatase inhibitors (Pierce) and were sonicated briefly for 30 s. Lysates were cleared by centrifugation at 14,000*g* for 10 min, and protein concentration was determined using the BCA kit (Pierce), which measures the reduction of Cu2+ to Cu1+ by protein in an alkaline medium. For each sample, 25 µg of protein lysate was loaded onto 4–12% SDS-PAGE minigels (Invitrogen) for electrophoresis and immunoblotting against H3K4me1 (Cell Signaling 5326), H3K4m3 (Cell Signaling 9727), Histone H3 (Cell Signaling 9715), ERα (Santa Cruz 7207), pERα S118 (Signalway Antibody 11072), pERα S167 (Cell Signaling 5587), pERα S104/106 (Cell Signaling 2517), PR (Cell Signaling 8757), β-actin (Cell Signaling 4970).

### Quantitative RT-PCR

RNA was extracted from cells 48 h after transfection or from the mammary glands of 12-week old virgin females using the RNeasy Mini kit (Qiagen) according to the manufacturer’s protocol. cDNA was synthesized with 1 µg of RNA from each sample using the qScript cDNA SuperMix (Quanta Biosciences) according to the manufacturer’s protocol. Synthesized cDNA was diluted with one volume of DEPC-treated water, and 2 µl of the mixture was added to TaqMan PCR Master Mix (Applied Biosystems) along with primers. Relative quantification for each mRNA was performed using the comparative CT method with a ViiA 7 Real-Time PCR system (Applied Biosystems)32. Samples were run in triplicate, and mRNA levels were normalized to those of *RPLP0* or *Gapdh* for each reaction. TaqMan primers were all purchased from Applied Biosystems. Catalog numbers are as follows: *AGR3* (Hs00411286_m1), *BANK1* (Hs01009378_m1), *CA2* (Hs01070108_m1), *CACNA1G* (Hs00367969_m1), *CAV1* (Hs00971716_m1), *CD44* (Hs01075861_m1), *CXCL12* (Hs03676656_mH), *ESR1* (Hs00174860_m1), *GDNF* (Hs01931883_s1), *IGFBP4* (Hs01057900_m1), *KMT2A* (Hs00610538_m1), *KMT2B* (Hs00207065_m1), *KMT2C* (Hs01005521_m1), *KMT2D* (Hs00231606_m1), *KRT13* (Hs02558881_s1), *PEG10* (Hs00248288_s1), *PGR* (Hs01556707_m1), *RPLP0* (Hs99999902_m1), *SERPINA1* (Hs00165475_m1), *SOX5* (Hs00753050_s1), *TFF3* (Hs00902278_m1), *TMPRSS3* (Hs00917537_m1), *Kmt2c* (Mm01156942_m1), *Pgr* (Mm00435628_m1), *Gapdh* (4331182).

### Chromatin Immunoprecipitation

Complexes were purified using H3K4me1 (abcam 8895), H3K27ac (abcam 4729), and ERα (sc-453) -specific antibodies and DNA was purified using the PCR Purification Kit (Qiagen). ChIP primers are as follows *CA2* enhancer forward: 5′-GCAATCCTGAGAAACTGCAA-3′, reverse: 5′-GCTCCTGGCCTCAAACTATC-3′, *PGR* enhancer forward: 5′- GAGGGCTGTTGAAACAAACA-3′, reverse: 5′- TGCTGAGATCACACCTAGACAA-3′, *AGR2* enhancer forward: 5′-ACGAAGCCTGCTT CTGAAC-3′, reverse: 5′-GTTTACAAGACATCAAACAACATGA-3′. IRX4 enhancer forward: 5′- AGGGATCACTCCAGACTCCT-3′, reverse: 5′- CTGAGTGCCAGATGTGCTTC-3′. MUC1 enhancer forward: 5′- TCCTCGAGAAGAGCAACTCC-3′, reverse: 5′- CCGAGAGATCCGCTGTTAGT-3′. CMYC enhancer forward: 5′- AAGACTGCCTCCCGCTTTGT-3′, reverse: 5′- TGCTGCTGCTGCTGGTAGAA-3′.

### Immunohistochemistry

Tissue were fixed in 10% neutral buffered formalin for 48 h, were processed in xylene and ethanol, embedded in paraffin, sectioned at 4 µm thickness, and stained with hematoxylin and eosin or with an antibody against the PR (Santa Cruz sc-538) and then detected with SignalStain Boost IHC Detection Reagent (Cell Signaling, 8114). Slides were examined by a board-certified pathologist (S. Monette, MSKCC).

### Rapid immunoprecipitation mass spectrometry of endogenous proteins (RIME)

RIME was performed as previously described [21, 22]. Briefly, MCF7 cells were grown in SILAC Protein Quantification Kit DMEM:F12 (Thermo Fischer Scientific 88439) supplemented with dialyzed FBS (Life Technologies 88440) with either l-Lysine-2HCl (Thermo Scientific 88429) and l-Arginine-HCl (Thermo Scientific 88427) for light-labeled media or l-Arginine-HCl 13C6 (Cambridge Isotope Laboratories 13E-430) and l-Lysine-2HCl 13C6, 15N2 (Cambridge Isotope Laboratories 14F-832) for heavy-labeled media. A total of 60 million cells total with 30 million light labeled shRenilla cells and 30 million heavy labeled shKMT2C#2 cells was used for each replicate. Ten micrograms of antibody against ERα (Santa Cruz sc-543) were used for each replicate. There were three replicates done in total.

Immunoprecipitated samples were washed as previously described and digested overnight with trypsin, peptides desalted using C18 zip tips, and then dried by vacuum centrifugation. Each sample was reconstituted in 10 µL 0.1% (vol/vol) formic acid and 4 µL analyzed by microcapillary liquid chromatography with tandem mass spectrometry using the NanoAcquity (Waters) with a 100-μm-inner-diameter × 10-cm- length C18 column (1.7 um BEH130, Waters) configured with a 180-μm × 2-cm trap column coupled to a Q-Exactive Plus mass spectrometer (Thermo Fisher Scientific). Peptides were eluted with a linear gradient of 0–35% acetonitrile (0.1% formic acid) in water (0.1% formic acid) over 150 mins with a flow rate of 300 nL/min. The QE Plus was operated in automatic, data-dependent MS/MS acquisition mode with one MS full scan (380–1800 *m/z*) at 70,000 mass resolution and up to ten concurrent MS/MS scans for the ten most intense peaks selected from each survey scan. Survey scans were acquired in profile mode and MS/MS scans were acquired in centroid mode at 17,500 resolution and isolation window of 1.5 amu and normalized collision energy of 27. AGC was set to 1 × 10 [6] for MS1 and 5 × 10 [4] and 100 ms IT for MS2. Charge exclusion of unassigned and greater than 6 enabled with dynamic exclusion of 15 s. All MS/MS samples were analyzed using MaxQuant (Max Planck Institute of Biochemistry, Martinsried, Germany; version 1.5.3.3) at default settings with a few modifications. The default was used for first search tolerance and main search tolerance: 20 and 4.5 ppm, respectively. Labels were set to Arg6 and Lys8. MaxQuant was set up to search the reference human proteome database downloaded from Uniprot on September 6, 2016. Maxquant performed the search assuming trypsin digestion with up to two missed cleavages. Peptide, Site, and Protein FDR were all set to 1%. One unique peptide was required for high-confidence protein identifications and a minimum ratio count of two peptides (one unique and one razor) were required for SILAC ratio determination. The following modifications were used as variable modifications for identifications and included for protein quantification: oxidation of methionine, acetylation of the protein N-terminus, phosphorylation of serine, threonine and tyrosine residues.

### Sequencing

RNA was extracted from MCF7 cells stably expressing shRenilla, shKMT2C #1 and shKMT2C #2 using the RNeasy Mini kit (Qiagen) according to the manufacturer’s protocol. RNA size, concentration and integrity were analyzed using Agilent 2100 Bioanalyzer. Libraries were generated using Illumina’s TruSeq RNA sample Prep Kit v2, following the maufacturer’s protocol. Sequencing was done on the HiSeq2500 sequencer as 50-bp paired-read with 30–40 million reads per sample by the NYU Genome Technology Center.

The raw data were analyzed using Basepair software (http://www.basepairtech.com/) with pipelines including the following steps: raw reads were aligned to the transcriptome derived from UCSC genome assembly hg19 using STAR [53] with default parameters. Read counts for each transcript were measured using featureCounts [54]. Differentially expressed genes were determined using DESeq2 [55] and a cut-off of 0.05 on adjusted *p*-value (corrected for multiple hypotheses testing) was used for creating lists and heatmaps, unless otherwise stated.

### Chromatin Immunoprecipitation followed by sequencing

A total of 2 × 10^6^ MCF7 cells stably expressing either shRenilla, shKMT2C #1 or shKMT2C 2 were fixed with 1% formaldehyde at room temperature, lysed and sonicated leading to a DNA average size of 200 bp. 1ug of H3K4me1 (abcam 8895), H3K27ac (abcam 4729) and ERα (Santa Cruz HC-20)-specific antibodies was added to the samples and incubated overnight at 4 °C. The complexes were purified using ChIP grade protein-A/G magnetic beads (Thermo Scientific QB210263) followed by elution from the beads and reverse cross-linking. DNA was purified using PCR purification columns (QIAGEN). H3K4me1, H3K4me3 and ERα ChIP-seq libraries were prepared using 5ng of DNA and KAPA Hyper Prep Kit for Illumina, according to the manufacturer’s protocol. Libraries were validated using the Agilent 2200 Tapestation and KAPA library quantification qPCR assay and sequenced on a HiSeq2500 sequencer as 50-bp single-end reads by the NYU Genome Technology Center.

The raw data were analyzed using Basepair software (http://www.basepairtech.com/) with pipelines including the following steps: The raw fastq data were trimmed using trim_galore to remove low-quality ends from reads (quality <15) and adapter sequences. The trimmed data was aligned using Bowtie2 [56] to UCSC genome assembly hg19. Duplicate reads were removed using Picard and bigwig files were created for visualization. Peaks were identified with Macs1.4 [57] and transcription factor binding motifs were detected with Homer. Motifs were identified as enriched over 48,246 randomly selected background sequences with matched %GC content. Peaks overlapping with Satellite repeat regions were discarded and remaining filtered peaks were annotated using custom scripts based on UCSC Flat data [58], where peaks between −2500 and 2500 bp of a transcription start site were marked as Promoter, the overlapping gene body were marked as Genebody and the rest were marked as Intergenic. For intergenic peaks, a gene was considered a target if it was within 1 Mb of the peak.

### Generation of conditional KMT2C knockout mice

Mice with the *Kmt2c* floxed (F) allele were generated by Biocytogen (Worcester, MA) in a C57BL/6 background. ES cells were verified using Southern blotting. F/F mice were bred to transgenic mice mice expressing the Cre enzyme in the mammary tissue under the control of the MMTV long-terminal repeat (mice were a kind gift from S. Lowe). Cre-mediated excision of the Kmt2c gene was verified by genotyping PCR of DNA extracted from toe clips or mammary fat pads. Genotyping for the MMTV-cre transgenes were done using generic Cre primers, forward (oIMR1084): 5′-GCGGTCTGGCAGTAAAAACTATC-3′, reverse (oIMR1085): 5′-GTGAAACAGCATTGCTGTCACTT-3′, as well as internal positive controls, forward (oIMR7338): 5′-CTAGGCCACAGAATTGAAAGATCT-3, reverse (oIMR7339): 5′-GTAGGTGGAAATTCTAGCATCATCC-3′. KMT2C floxed allele was genotyped using A1 primers, forward: 5′-GCAGAATCAAGGAGCTGTCTG-3′, reverse: 5′-TGCCTTGAGGTCAACGTACAATTG-3′. Cre mediated excision of KMT2C exon 3 was verified by the presence of a 3′LoxP band at 372 bp using the following primers, forward: 5′-GGAGCTGTCTGTTCAAGTATTTAGC-3′, reverse: 5′-GTGTGCCTAGGTATCCACAGAG-3′. For the in vivo experiments, 12-week were used. All mice were housed in the MSKCC animal facility.

### METABRIC [26] patients and tumor samples

A total of 1209 Luminal A and B breast cancer patients with Illumina HumanHT-12 V3 expression beadchip array and complete clinical annotation from the METABRIC data set were included in the analysis.

### Scoring for shKMT2C signatures with ssGSEA in primary METABRIC patients

ssGSEA [2] was used to compute a set of different scores based on in vitro-derived shKMT2C expression signatures. For each sample independently, an ssGSEA score was calculated after gene expression levels were rank normalized and ordered, using the empirical cumulative distribution functions of genes in the signature and the reaming genes as previously described [1, 2, 7] using the bioconductor package GSVA [6]. Patients were stratified into high, low and intermediate scorers using population quartiles for the numerical ssGSEA score.

### Study Population, clinical annotation and prospective sequencing for patients with aromatase inhibition treatment

A total of 671 breast tumor specimens from 466 patients with ER+/HER2− metastatic breast cancer who received aromatase inhibitors in metastatic setting underwent prospective clinical genomic profiling between April 2014 and March 2017. For each treatment line, the time of biopsy collection for MSK-IMPACT testing was compared with the treatment start and stop dates. We excluded samples that were collected after the start of CDK4/6 inhibitor therapy (*n* = 263 samples) and only included samples that were collected prior of therapy initiation. For the patients with multiple sequenced specimens we only included one pre-treatment sample and selected the sample that was collected within a shorter time interval prior to initiation of aromatase inhibitor therapy. The final cohort included 347 pre-treatment samples from 347 patients.

For all 347 patients, tumor and patient-matched normal DNA samples were extracted from either representative formalin-fixed paraffin embedded tumor biopsy samples or mononuclear cells from peripheral blood. All specimens underwent massively parallel next-generation sequencing in a CLIA-certified FDA-approved laboratory using MSK-IMPACT, a hybridization capture-based next-generation sequencing assay, which analyzes all protein-coding exons and selected intronic and regulatory regions of 341 to 468 cancer-associated genes, all as previously described [4, 21]. Somatic mutations, DNA copy number alterations, and structural rearrangements were identified as previously described [21] and all mutations were manually reviewed. The oncogenic *KMT2C* alterations were assessed using the latest versions of the OncoKB [5] [www.oncokb.org] and cancer hotspots [6] [www.cancerhotspots.org] knowledge bases and only alterations that are considered “oncogenic” or “likley oncogenic” were included in the analysis.

### Generation of MCF7 KMT2C CRISPR by CRISPR/Cas9nickase

The following guide RNA sequences targeting KMT2C exon 6 were selected using the Optimized CRISPR Design tool (http://crispr.mit.edu [51]): gRNA1 CTAGTGACCACTCCA CACAACGG, gRNA2 AGAACCATTGTTAGTGAACGTGG.

DNA oligonucleotides were purchased from IDT and cloned into pX335-GFP vector [52] to generate targeting constructs that were subsequently co-transfected in an equimolar ratio into MCF7 cells using Lipofectamine HP (Thermo Fischer Scientific). Seventy-two hours after transfection, cells were sorted using a MoFlo cell sorter (Beckman Coulter) for cells expressing Cas9 nickase (GFP-positive cells) and left to recover for 1 week before sorting for single cells and allowing colonies to form. Indels within the KMT2C exon 6 were detected using the Surveyor Mutation Detection kit (IDT) and confirmed by subcloning and sequencing of KMT2C exon 6. One clone showing knockout of all 2/3 KMT2C alleles was used for subsequent analysis.

### Statistics

Patient data was collected retrospectively; therefore, we did not predetermine a sample size. Sample size estimates for animal studies were determined based on previous reports using similar sample sizes [59]. Samples were placed into their experimental groups according to their type, and so randomization was not used. Investigators were not blinded to the experimental groups. Only female mice were considered as predetermined by the fact that we were interested in phenotypic effects to the mammary gland. qPCR data and qChIP were obtained from independent technical replicates (*n* values indicated in the corresponding figure legends). Normal distribution and equal variance was seen in the majority of data and, so, we assumed normality and equal variance for all samples. On this basis, we used the Student’s *t*-test (two-tailed, unpaired) to estimate statistical significance. Analysis was conducted using Prism v7.0c (GraphPad Software). Survival for the METABRIC patient data was reported using Kaplan–Meier curves, and significance was estimated with the log-rank test. Statistical analysis for survival curves of patients with aromatase inhibitor treatment used univariate Cox proportional hazard models to determine the association between oncogenic *KMT2C* alterations and PFS with disease progression on aromatase inhibitors or patient death. For patients with multiple lines of therapy, only the first treatment line from that class that was started after the MSK-IMPACT biopsy was included in the analysis. We tested the proportionality assumption of the Cox regression model through time-dependency analysis of selected genetic alterations (cox.zph function of the R package survival). We rejected the null hypotheses with a two-sided α = 0.05. Statistical significance was determined by log-rank test stratified by the treatment.

### Study approval

All mice were treated with procedures approved by the Institutional Animal Care and Use Committee. Access to the METABRIC [26] cohort was obtained with consent from the MSKCC institutional review board for anonymized evaluation of genomic associations. Study involving patients treated with aromatase inhibitors was approved by the Memorial Sloan Kettering Cancer Center Institutional Review Board and all patients provided written informed consent for tumor sequencing and review of patient medical records for detailed demographic, pathologic, and treatment information (NCT01775072). Detailed treatment history data were obtained for each patient and included all lines of systemic therapy from time of diagnosis of invasive carcinoma to the study data lock in May 2017. The exact regimen as well as the dates of start and stop of therapy were recorded for each treatment line.

### Data deposition

Our ChIP sequencing and RNA sequencing data has been deposited in NCBI GEO

Accession number: GSE100328.

## Electronic supplementary material

Supplemental Results and Methods

Supplemental Table 1

Supplemental Table 2

Supplemental Table 3

## References

[1] Flockhart RJ, Webster DE, Qu K, Mascarenhas N, Kovalski J, Kretz M (2012). BRAFV600E remodels the melanocyte transcriptome and induces BANCR to regulate melanoma cell migration. Genome Res.

[2] Kannengiesser C, Spatz A, Michiels S, Eychene A, Dessen P, Lazar V (2008). Gene expression signature associated with BRAF mutations in human primary cutaneous melanomas. Mol Oncol.

[3] Guo X, Xu Y, Zhao Z (2015). In-depth genomic data analyses revealed complex transcriptional and epigenetic dysregulations of BRAFV600E in melanoma. Mol Cancer.

[4] Wellbrock C, Rana S, Paterson H, Pickersgill H, Brummelkamp T, Marais R (2008). Oncogenic BRAF regulates melanoma proliferation through the lineage specific factor MITF. PLoS One.

[5] Kim J, Woo AJ, Chu J, Snow JW, Fujiwara Y, Kim CG (2010). A Myc network accounts for similarities between embryonic stem and cancer cell transcription programs. Cell.

[6] Richart L, Carrillo-de Santa Pau E, Rio-Machin A, de Andres MP, Cigudosa JC, Lobo VJ (2016). BPTF is required for c-MYC transcriptional activity and in vivo tumorigenesis. Nat Commun.

[7] Richart L, Real FX, Sanchez-Arevalo Lobo VJ (2016). c-MYC partners with BPTF in human cancer. Mol Cell Oncol.

[8] Calo E, Wysocka J (2013). Modification of enhancer chromatin: what, how, and why?. Mol Cell.

[9] Kandoth C, McLellan MD, Vandin F, Ye K, Niu B, Lu C (2013). Mutational landscape and significance across 12 major cancer types. Nature.

[10] Ciriello G, Gatza ML, Beck AH, Wilkerson MD, Rhie SK, Pastore A (2015). Comprehensive molecular portraits of invasive lobular breast. Cancer Cell.

[11] Ellis MJ, Ding L, Shen D, Luo J, Suman VJ, Wallis JW (2012). Whole-genome analysis informs breast cancer response to aromatase inhibition. Nature.

[12] Lee JE, Wang C, Xu S, Cho YW, Wang L, Feng X (2013). H3K4 mono- and di-methyltransferase MLL4 is required for enhancer activation during cell differentiation. eLife.

[13] Kaikkonen MU, Spann NJ, Heinz S, Romanoski CE, Allison KA, Stender JD (2013). Remodeling of the enhancer landscape during macrophage activation is coupled to enhancer transcription. Mol Cell.

[14] Fellmann C, Hoffmann T, Sridhar V, Hopfgartner B, Muhar M, Roth M (2013). An optimized microRNA backbone for effective single-copy RNAi. Cell Rep.

[15] Hurtado A, Holmes KA, Ross-Innes CS, Schmidt D, Carroll JS (2011). FOXA1 is a key determinant of estrogen receptor function and endocrine response. Nat Genet.

[16] Valekunja UK, Edgar RS, Oklejewicz M, van der Horst GT, O’Neill JS, Tamanini F (2013). Histone methyltransferase MLL3 contributes to genome-scale circadian transcription. Proc Natl Acad Sci USA.

[17] Creyghton MP, Cheng AW, Welstead GG, Kooistra T, Carey BW, Steine EJ (2010). Histone H3K27ac separates active from poised enhancers and predicts developmental state. Proc Natl Acad Sci USA.

[18] Lupien M, Eeckhoute J, Meyer CA, Wang Q, Zhang Y, Li W (2008). FoxA1 translates epigenetic signatures into enhancer-driven lineage-specific transcription. Cell.

[19] Joseph R, Orlov YL, Huss M, Sun W, Kong SL, Ukil L (2010). Integrative model of genomic factors for determining binding site selection by estrogen receptor-alpha. Mol Syst Biol.

[20] Carroll JS, Meyer CA, Song J, Li W, Geistlinger TR, Eeckhoute J (2006). Genome-wide analysis of estrogen receptor binding sites. Nat Genet.

[21] Mohammed H, Taylor C, Brown GD, Papachristou EK, Carroll JS, D’Santos CS (2016). Rapid immunoprecipitation mass spectrometry of endogenous proteins (RIME) for analysis of chromatin complexes. Nat Protoc.

[22] Mohammed H, Russell IA, Stark R, Rueda OM, Hickey TE, Tarulli GA (2015). Progesterone receptor modulates ERalpha action in breast cancer. Nature.

[23] Mohammed H, D’Santos C, Serandour AA, Ali HR, Brown GD, Atkins A (2013). Endogenous purification reveals GREB1 as a key estrogen receptor regulatory factor. Cell Rep.

[24] Masamura S, Santner SJ, Heitjan DF, Santen RJ (1995). Estrogen deprivation causes estradiol hypersensitivity in human breast cancer cells. J Clin Endocrinol Metab.

[25] Jeng MH, Shupnik MA, Bender TP, Westin EH, Bandyopadhyay D, Kumar R (1998). Estrogen receptor expression and function in long-term estrogen-deprived human breast cancer cells. Endocrinology.

[26] Curtis C, Shah SP, Chin SF, Turashvili G, Rueda OM, Dunning MJ (2012). The genomic and transcriptomic architecture of 2,000 breast tumours reveals novel subgroups. Nature.

[27] Dumont JA, Bitonti AJ, Wallace CD, Baumann RJ, Cashman EA, Cross-Doersen DE (1996). Progression of MCF-7 breast cancer cells to antiestrogen-resistant phenotype is accompanied by elevated levels of AP-1 DNA-binding activity. Cell Growth Differ.

[28] Johnston SR, Lu B, Scott GK, Kushner PJ, Smith IE, Dowsett M (1999). Increased activator protein-1 DNA binding and c-Jun NH2-terminal kinase activity in human breast tumors with acquired tamoxifen resistance. Clin Cancer Res.

[29] Malorni L, Giuliano M, Migliaccio I, Wang T, Creighton CJ, Lupien M (2016). Blockade of AP-1 potentiates endocrine therapy and overcomes resistance. Mol Cancer Res.

[30] Paech K, Webb P, Kuiper GG, Nilsson S, Gustafsson J, Kushner PJ (1997). Differential ligand activation of estrogen receptors ERalpha and ERbeta at AP1 sites. Science.

[31] Schiff R, Reddy P, Ahotupa M, Coronado-Heinsohn E, Grim M, Hilsenbeck SG (2000). Oxidative stress and AP-1 activity in tamoxifen-resistant breast tumors in vivo. J Natl Cancer Inst.

[32] Zhou Y, Yau C, Gray JW, Chew K, Dairkee SH, Moore DH (2007). Enhanced NF kappa B and AP-1 transcriptional activity associated with antiestrogen resistant breast cancer. BMC Cancer.

[33] Gutierrez MC, Detre S, Johnston S, Mohsin SK, Shou J, Allred DC (2005). Molecular changes in tamoxifen-resistant breast cancer: relationship between estrogen receptor, HER-2, and p38 mitogen-activated protein kinase. J Clin Oncol.

[34] Wang C, Mayer JA, Mazumdar A, Fertuck K, Kim H, Brown M (2011). Estrogen induces c-myc gene expression via an upstream enhancer activated by the estrogen receptor and the AP-1 transcription factor. Mol Endocrinol.

[35] Lee S, Lee J, Lee SK, Lee JW (2008). Activating signal cointegrator-2 is an essential adaptor to recruit histone H3 lysine 4 methyltransferases MLL3 and MLL4 to the liver X receptors. Mol Endocrinol.

[36] Goo YH, Sohn YC, Kim DH, Kim SW, Kang MJ, Jung DJ (2003). Activating signal cointegrator 2 belongs to a novel steady-state complex that contains a subset of trithorax group proteins. Mol Cell Biol.

[37] Bhan A, Hussain I, Ansari KI, Kasiri S, Bashyal A, Mandal SS (2013). Antisense transcript long noncoding RNA (lncRNA) HOTAIR is transcriptionally induced by estradiol. J Mol Biol.

[38] Kim DH, Lee J, Lee B, Lee JW (2009). ASCOM controls farnesoid X receptor transactivation through its associated histone H3 lysine 4 methyltransferase activity. Mol Endocrinol.

[39] Lee S, Kim DH, Goo YH, Lee YC, Lee SK, Lee JW (2009). Crucial roles for interactions between MLL3/4 and INI1 in nuclear receptor transactivation. Mol Endocrinol.

[40] Kim DH, Rhee JC, Yeo S, Shen R, Lee SK, Lee JW (2015). Crucial roles of mixed-lineage leukemia 3 and 4 as epigenetic switches of the hepatic circadian clock controlling bile acid homeostasis in mice. Hepatology.

[41] Ananthanarayanan M, Li Y, Surapureddi S, Balasubramaniyan N, Ahn J, Goldstein JA (2011). Histone H3K4 trimethylation by MLL3 as part of ASCOM complex is critical for NR activation of bile acid transporter genes and is downregulated in cholestasis. Am J Physiol Gastrointest Liver Physiol.

[42] Ansari KI, Shrestha B, Hussain I, Kasiri S, Mandal SS (2011). Histone methylases MLL1 and MLL3 coordinate with estrogen receptors in estrogen-mediated HOXB9 expression. Biochemistry.

[43] Ansari KI, Hussain I, Shrestha B, Kasiri S, Mandal SS (2011). HOXC6 Is transcriptionally regulated via coordination of MLL histone methylase and estrogen receptor in an estrogen environment. J Mol Biol.

[44] Ansari KI, Hussain I, Kasiri S, Mandal SS (2012). HOXC10 is overexpressed in breast cancer and transcriptionally regulated by estrogen via involvement of histone methylases MLL3 and MLL4. J Mol Endocrinol.

[45] Lee J, Kim DH, Lee S, Yang QH, Lee DK, Lee SK (2009). A tumor suppressive coactivator complex of p53 containing ASC-2 and histone H3-lysine-4 methyltransferase MLL3 or its paralogue MLL4. Proc Natl Acad Sci USA.

[46] Chen C, Liu Y, Rappaport AR, Kitzing T, Schultz N, Zhao Z (2014). MLL3 is a haploinsufficient 7q tumor suppressor in acute myeloid leukemia. Cancer Cell.

[47] Zhang Z, Christin JR, Wang C, Ge K, Oktay MH, Guo W (2016). Mammary-stem-cell-based somatic mouse models reveal breast cancer drivers causing cell fate dysregulation. Cell Rep.

[48] Sato K, Akimoto K (2016). Expression levels of KMT2C and SLC20A1 identified by information-theoretical analysis are powerful prognostic biomarkers in estrogen receptor-positive breast cancer. Clin Breast Cancer.

[49] Won HH, Scott SN, Brannon AR, Shah RH, Berger MF (2013). Detecting somatic genetic alterations in tumor specimens by exon capture and massively parallel sequencing. J Vis Exp.

[50] Toy W, Shen Y, Won H, Green B, Sakr RA, Will M (2013). ESR1 ligand-binding domain mutations in hormone-resistant breast cancer. Nat Genet.

[51] Hsu PD, Scott DA, Weinstein JA, Ran FA, Konermann S, Agarwala V (2013). DNA targeting specificity of RNA-guided Cas9 nucleases. Nat Biotechnol.

[52] Cong L, Ran FA, Cox D, Lin S, Barretto R, Habib N (2013). Multiplex genome engineering using CRISPR/Cas systems. Science.

[53] Dobin A, Davis CA, Schlesinger F, Drenkow J, Zaleski C, Jha S (2013). STAR: ultrafast universal RNA-seq aligner. Bioinformatics.

[54] Liao Y, Smyth GK, Shi W (2014). featureCounts: an efficient general purpose program for assigning sequence reads to genomic features. Bioinformatics.

[55] Love MI, Huber W, Anders S (2014). Moderated estimation of fold change and dispersion for RNA-seq data with DESeq2. Genome Biol.

[56] Langmead B, Salzberg SL (2012). Fast gapped-read alignment with Bowtie 2. Nat Methods.

[57] Zhang Y, Liu T, Meyer CA, Eeckhoute J, Johnson DS, Bernstein BE (2008). Model-based analysis of ChIP-Seq (MACS). Genome Biol.

[58] Heinz S, Benner C, Spann N, Bertolino E, Lin YC, Laslo P (2010). Simple combinations of lineage-determining transcription factors prime cis-regulatory elements required for macrophage and B cell identities. Mol Cell.

[59] Feng Y, Manka D, Wagner KU, Khan SA (2007). Estrogen receptor-alpha expression in the mammary epithelium is required for ductal and alveolar morphogenesis in mice. Proc Natl Acad Sci USA.

[60] Razavi P, et al. (editors). Clinical genomic profiling of 1000 metastatic breast cancer patients: actionable targets, novel alterations, and clinical correlations. Cancer Res. 2016. Abstract 4509, AACR 107th Annual Meeting 2016.

