# Supplementary material for: KMT2C mediates the estrogen dependence of breast cancer through regulation of ERα enhancer function
Source: Oncogene. 2018 May 14;37(34):4692–710. doi: 10.1038/s41388-018-0273-5 (PMC6107480; doi:10.1038/s41388-018-0273-5)
Supplement: Supplementary file 1 — Supplemental Results and Methods [file 41388_2018_273_MOESM1_ESM.docx]

Supplementary Information


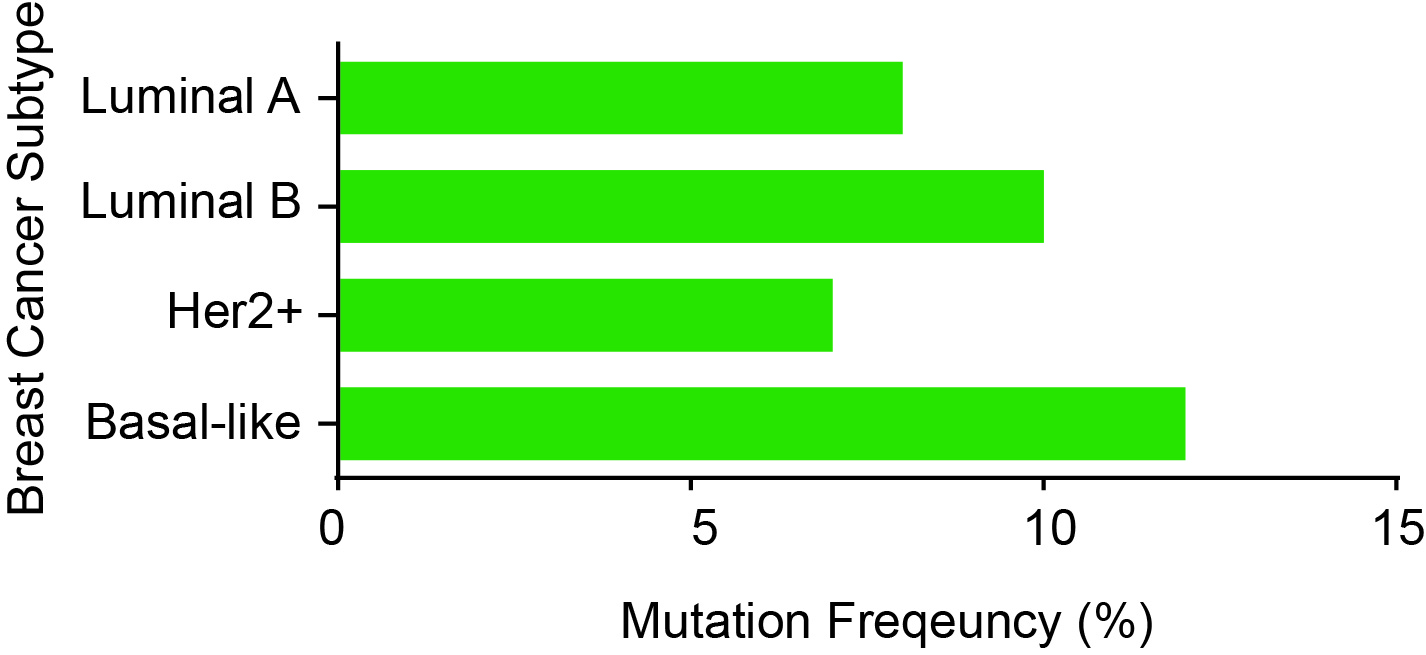


Supplementary Figure 1. KMT2C mutations are evenly distributed across breast cancer subtype. Percentage of KMT2C mutations across different breast cancer subtypes from the TCGA dataset(2) (n=818).

Supplementary Figure 2. KMT2C knockdown across breast cancer cell lines. (A) mRNA levels of indicated genes, as measured by qRT-PCR, in shRenilla, shKMT2C#1 and shKMT2C#2 MCF7 cells. Values correspond to the mean of three replicates ± s.e.m. (B) mRNA levels of *KMT2C*, as measured by qRT-PCR, in shRenilla, shKMT2C#1 and shKMT2C#2 T47D, CAMA-1, SKBR3, MDA-MB-468, HCC1806 and HCC1954 cells. Values correspond to the mean of three replicates ± s.e.m. Data correspond to one representative assay from a total of two or three independent assays. (C,D) Immunoblot of the indicated proteins in shRenilla or shKMT2C KMT2C-HA or Crispr control MCF7 cells cells. β-actin was used as a loading control.


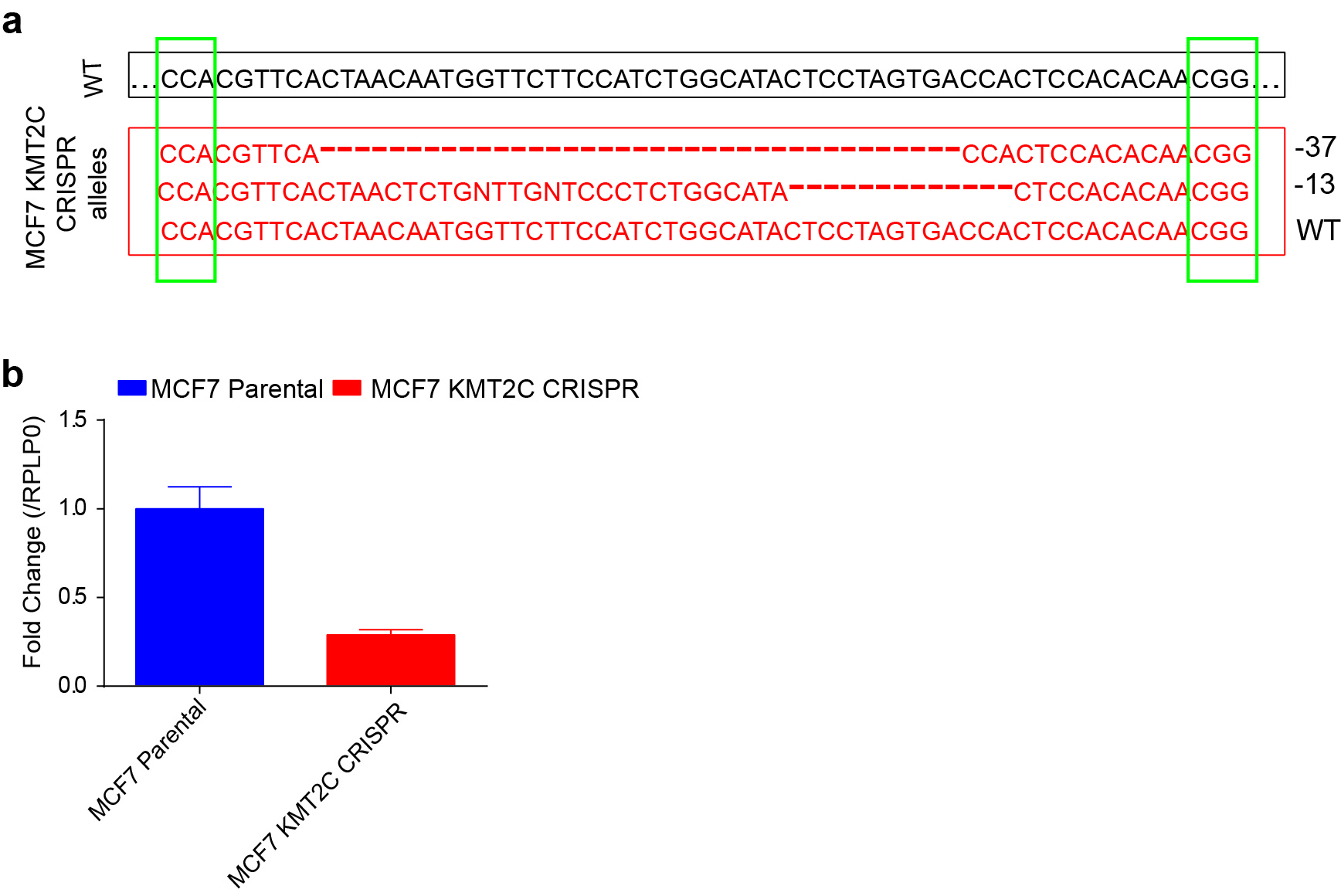


Supplementary Figure 3. Generation of MCF7 KMT2C crispr cells. (A) Mutant DNA sequences induced by *KMT2C* exon 6 sgRNAs in MCF7 (resulting cell line referred to as MCF7 KMT2C CRISPR cells). The PAM sequences are shown in the green outline and deleted nucleotides shown as dashes. WT, wild-type. MCF7 KMT2C CRISPR cells retained one WT copy as indicated. (B) mRNA levels, as measured by qRT-PCR, in parental and MCF7 KMT2C CRISPR cells. Values correspond to the mean of three technical replicates ± s.e.m. Data correspond to one representative assay from a total of three independent assays.


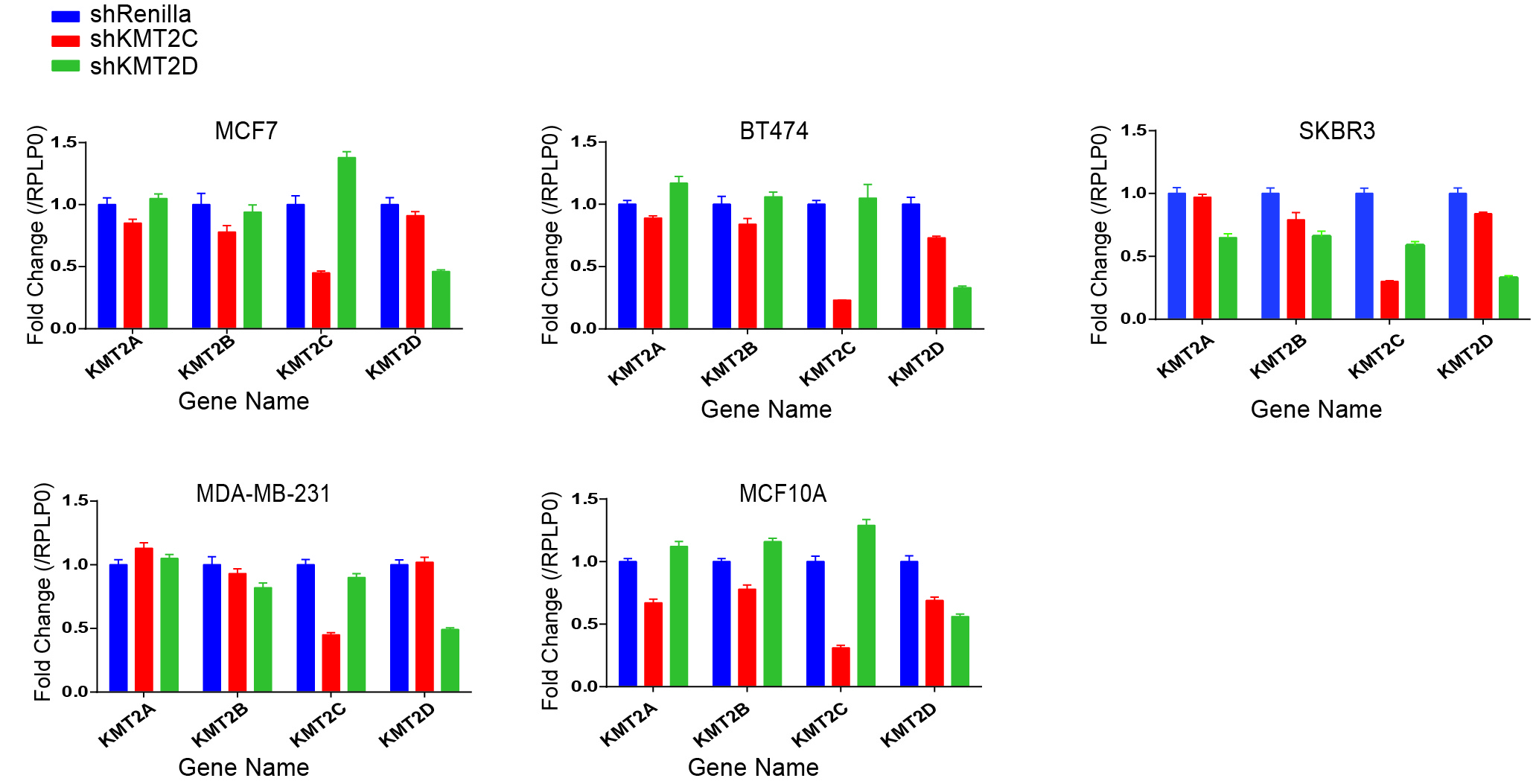


Supplementary Figure 4. KMT2C and KMT2D knockdown. mRNA levels of indicated genes, as measured by qRT-PCR, in the indicated shRenilla, shKMT2C and shKMT2D breast cancer cell lines. Values correspond to the mean of three replicates ± s.e.m.


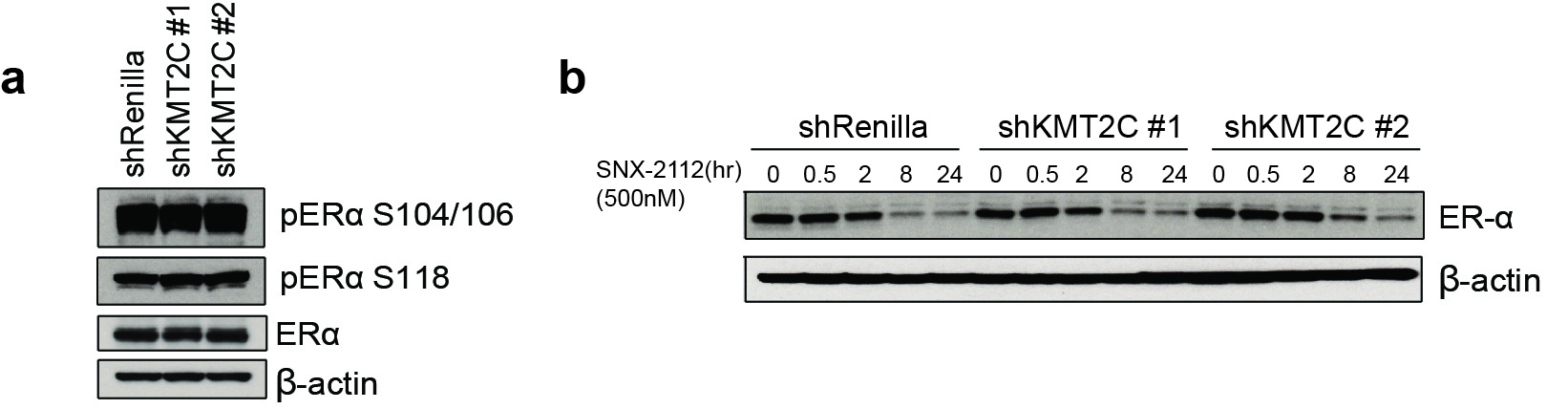


Supplementary Figure 5. Intrinsic ERα activity is unchanged following loss of KMT2C. (A,) Immunoblot of the indicated proteins in shRenilla or shKMT2C MCF7 cells. β-actin was used as a loading control. (B) shRenilla, shKMT2C#1 or shKMT2C#2 MCF7 cells were treated with HSP90 inhibitor, SNX2112 (500nmol/L), and collected at indicated times. Immunoblots of the indicated proteins are shown. β-actin was used as a loading control.


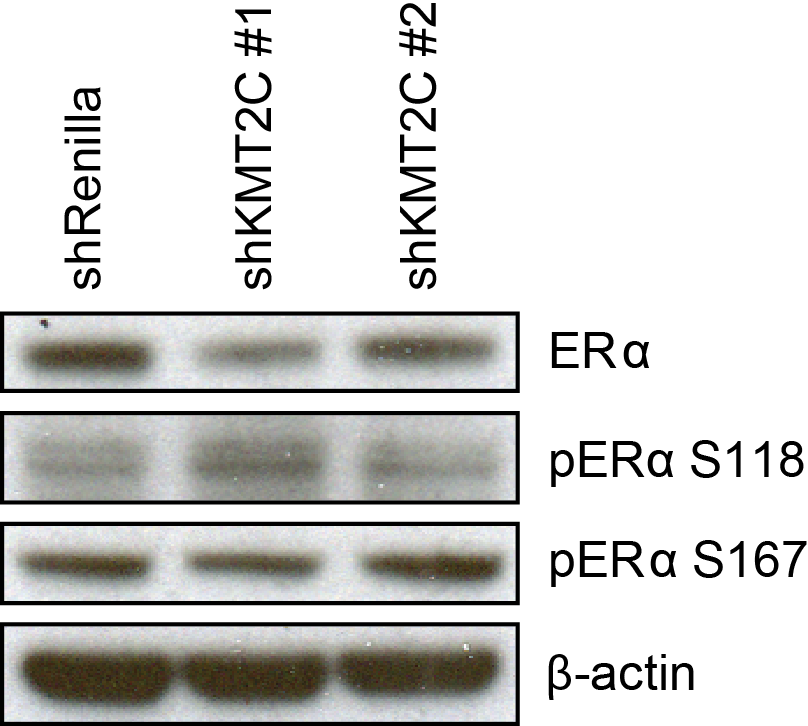


Supplementary Figure 6. ERα protein levels are unchanged following loss of KMT2C in T47D cells. Immunoblot of the indicated proteins in shRenilla or shKMT2C MCF7 cells. β-actin was used as a loading control.


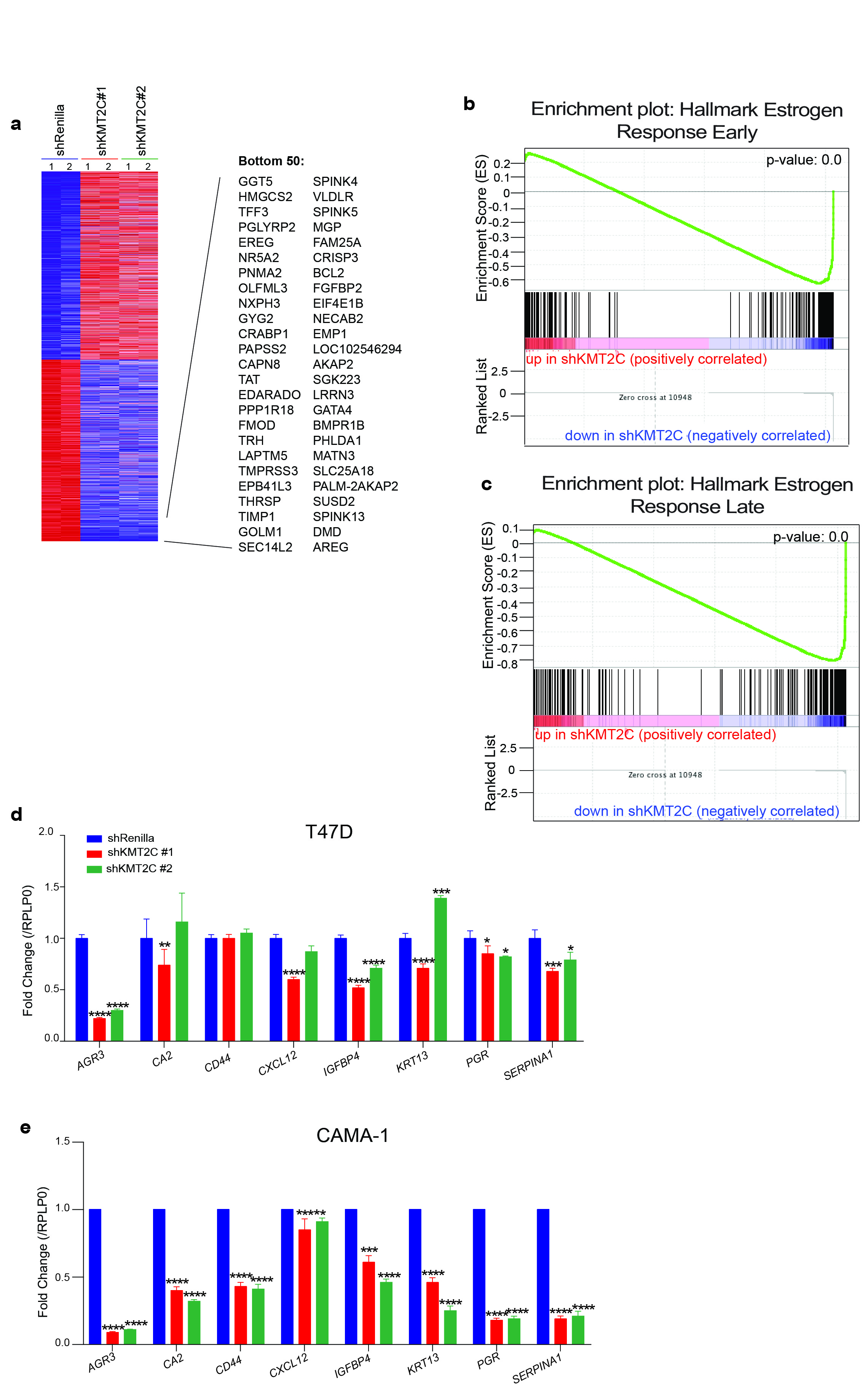


Supplementary Figure 7. Gene expression changes following KMT2C knockdown in T47D and Cama-1 cells. (A) Supervised analysis of the 4349 differentially expressed genes between T47D shRenilla and T47D shKMT2C (shKMT2C#1 and shKMT2C#5 samples combined). Columns represent individual replicates for shRenilla and shKMT2C samples, rows correspond to the different genes. Color reflects the normalized expression count (number of reads mapping to each gene); scaled by row. (B) GSEA of 2317 genes downregulated in shKMT2C as compared to the Hallmark Estrogen Response Early Geneset (Broad Institute). (C) GSEA of 2317 genes downregulated in shKMT2C as compared the Hallmark Estrogen Response Late Geneset. (D, E) mRNA levels, as measured by qRT-PCR, in T47D and CAMA-1 cells constitutively expressing either shRenilla, shKMT2C#1 or shKMT2C#2. Values correspond to the mean of three replicates ± s.e.m.; two-tailed Student’s *t*-test with a desired FDR = 1% was used to determine statistical significance; ***P* < 0.01, ****P* <0.001, *****P* <0.0001. Data correspond to one representative assay from a total of three independent assays.


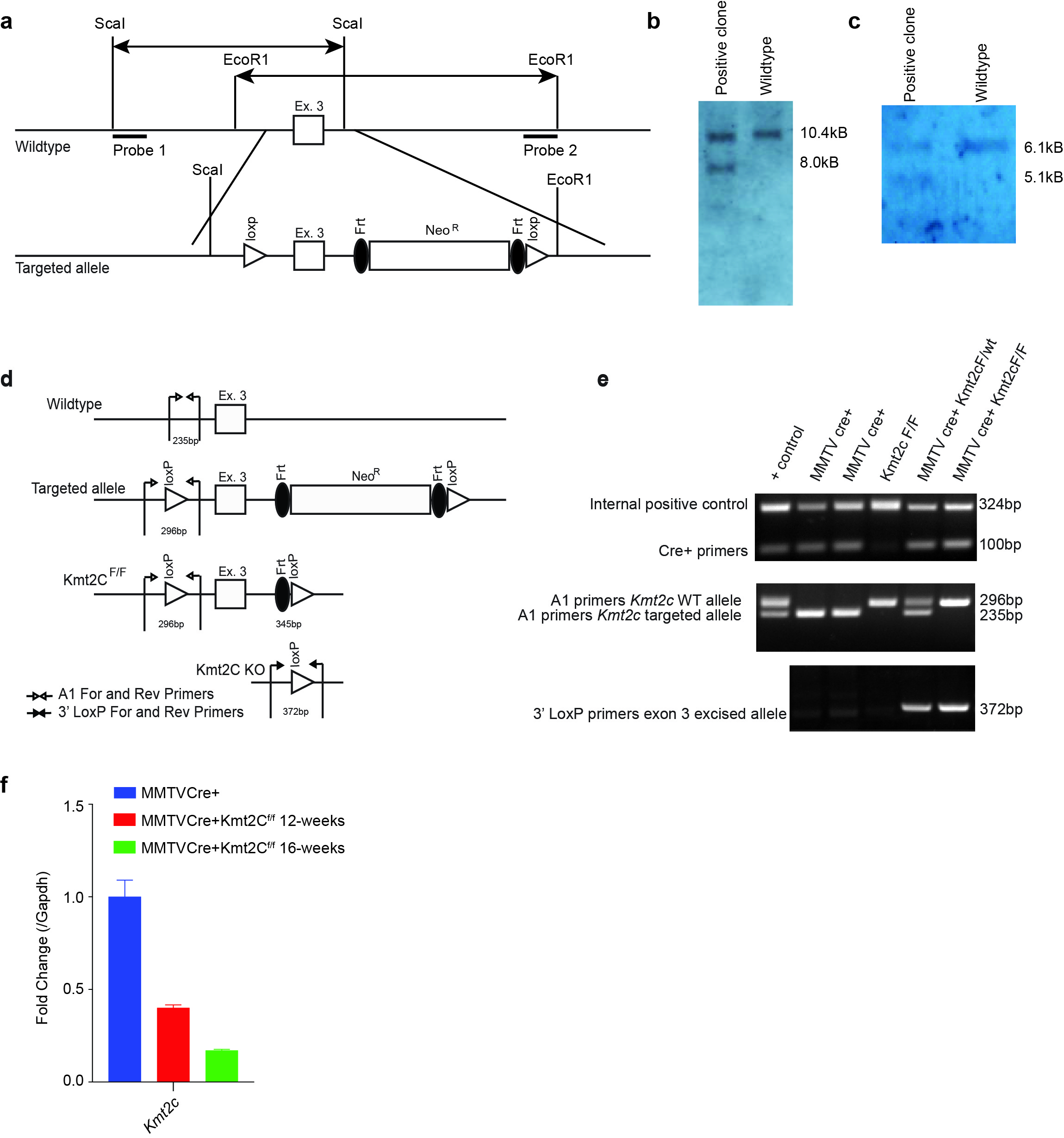


Supplementary Figure 8. Generation of *Kmt2c* floxed mice and loss of Kmt2c in MMTVCre+*Kmt2c*^f/f^ mice. (A) Diagram indicating components of the *Kmt2c* wildtype and targeted allele and the location of ScaI and EcoRI restriction enzyme sites and Southern blot probe recognition sites. (B) Southern blot with probe 1 following digestion with ScaI. Wildtype band is 10.4kB and targeted allele is 8.0kB (C) Southern blot with probe 2 following digestion with EcoR1. Wildtype band is 6.1kB and targeted allele is 5.1kB. (D) Targeted and conditional disruption of the mouse *Kmt2c* gene, using the Cre-loxP recombination system. Components of the *Kmt2c* exon 3 WT allele, the targeted allele after homologous recombination in ES cells, the floxed allele (*Kmt2c*^F/F^) after deletion of the neomycin resistance gene (pGK-Neo) in ES cells, and the deleted *Kmt2c* allele in Cre-recombinase transgenic mice (Kmt2cKO). (E) Genotyping mammary glands of virgin mice with the indicated genotypes with the positive control (+ control) being toe clips from an MMTVCre^+^*Kmt2c*^F/F^ mouse. (F) mRNA levels of indicated genes, as measured by qRT-PCR, from mammary glands of female mice of the indicated genotypes. Values correspond to the mean of three replicates ± s.e.m.


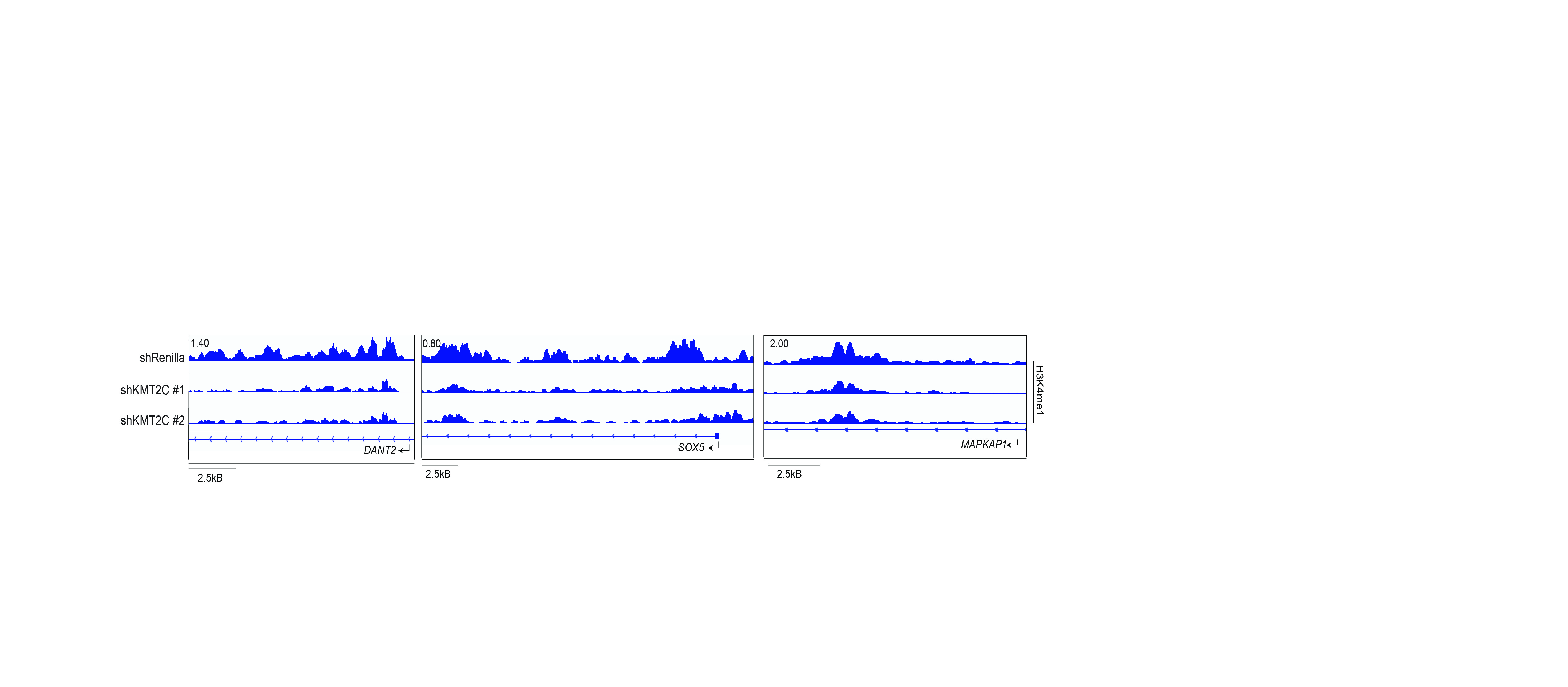


Supplementary Figure 9. H3K4me1 changes following KMT2C knockdown. IGV browser views for H3K4me1 in shRenilla, shKMT2C#1 and #2 MCF7 cells at sites of H3K4me1 loss.


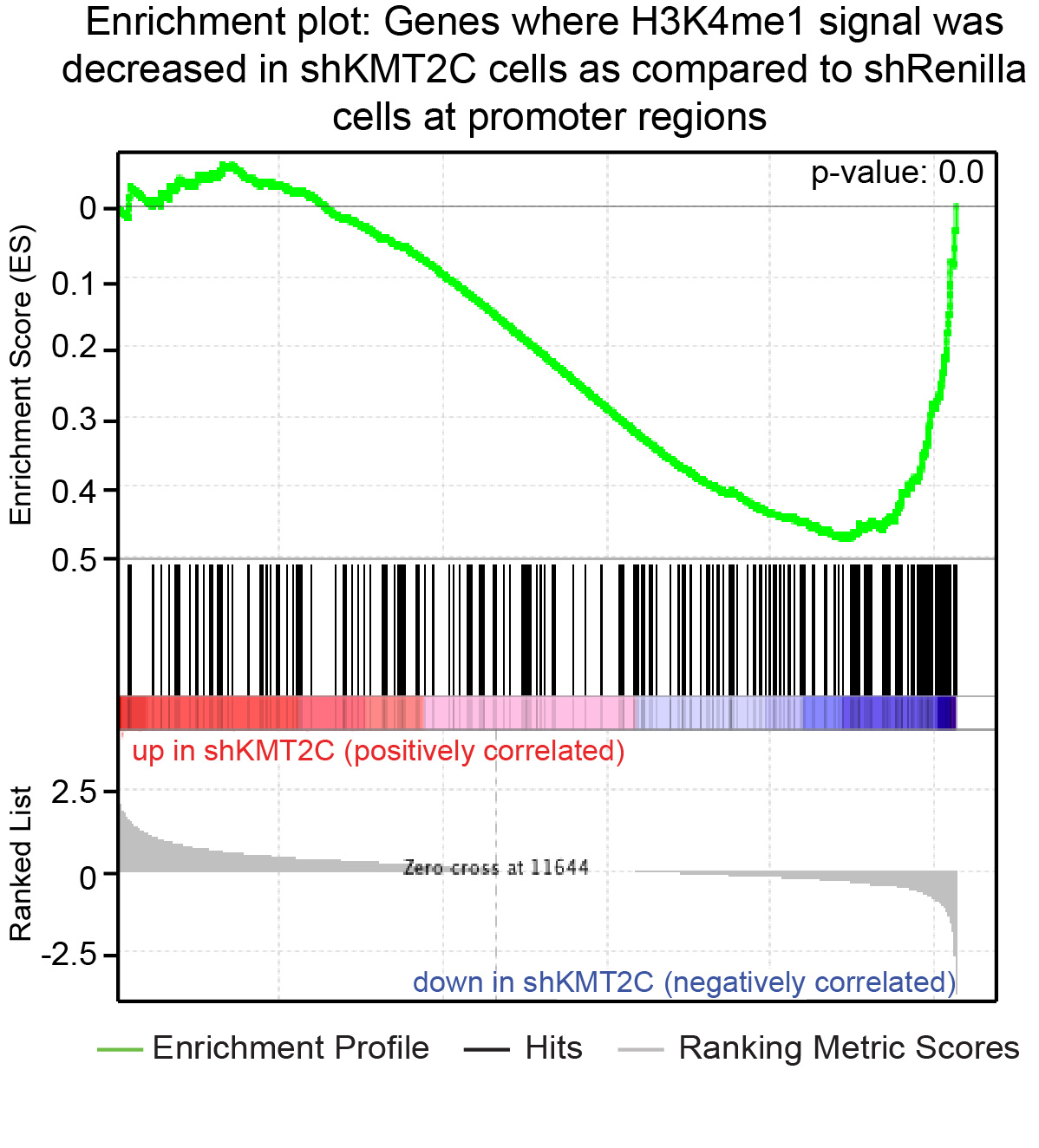


Supplementary Figure 10. H3K4me1 loss at promoter regions correlates with decreased gene expression. GSEA of 3857 genes significantly downregulated in MCF7 shKMT2C cells (p-val < 0.05) as compared to ranked genes with a ≥ 25% reduction in H3K4me1 marks at promoters in shKMT2C MCF7 cells.


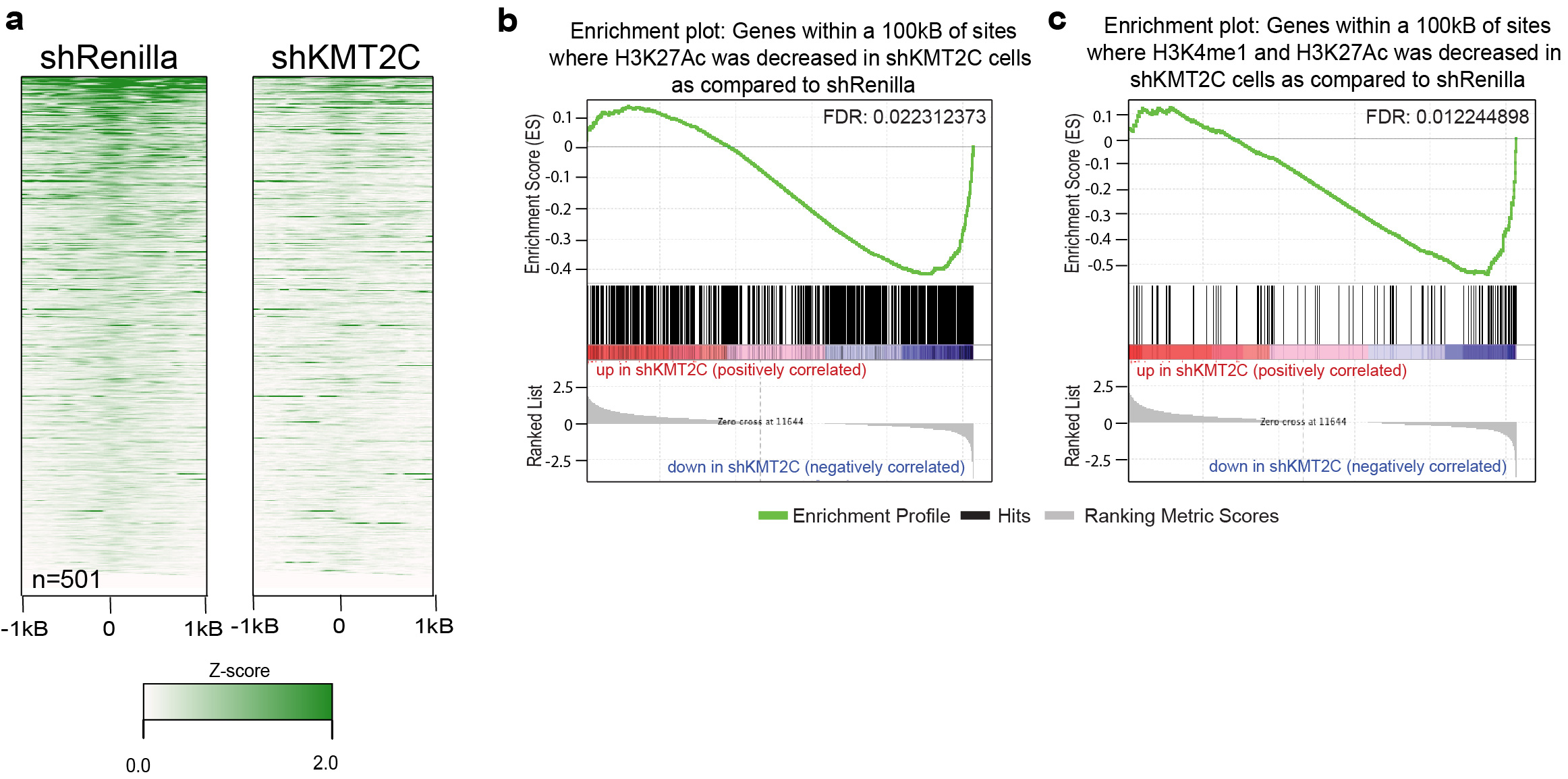


Supplementary Figure 11. Sites of H3K27ac loss in KMT2C knockdown cells correlate with reduced gene expression. (A) Normalized heatmaps for H3K27ac occupancy at sites of H3K4me1 loss in shRenilla, shKMT2C#1 and shKMT2C#2 cells among 501 sites. Heatmaps are centered at the H3K27ac peak summit. (B) GSEA of 3857 genes significantly downregulated in MCF7 shKMT2C cells (p-val < 0.05) as compared to ranked genes with a ≥ 25% reduction in H3K27ac marks at enhancers in shKMT2C MCF7 cells. (C) GSEA of 3857 genes significantly downregulated in MCF7 shKMT2C cells (p-val < 0.05) as compared to ranked genes with a ≥ 25% reduction in both H3K27ac and H3K4me1 marks at enhancers in shKMT2C MCF7 cells.


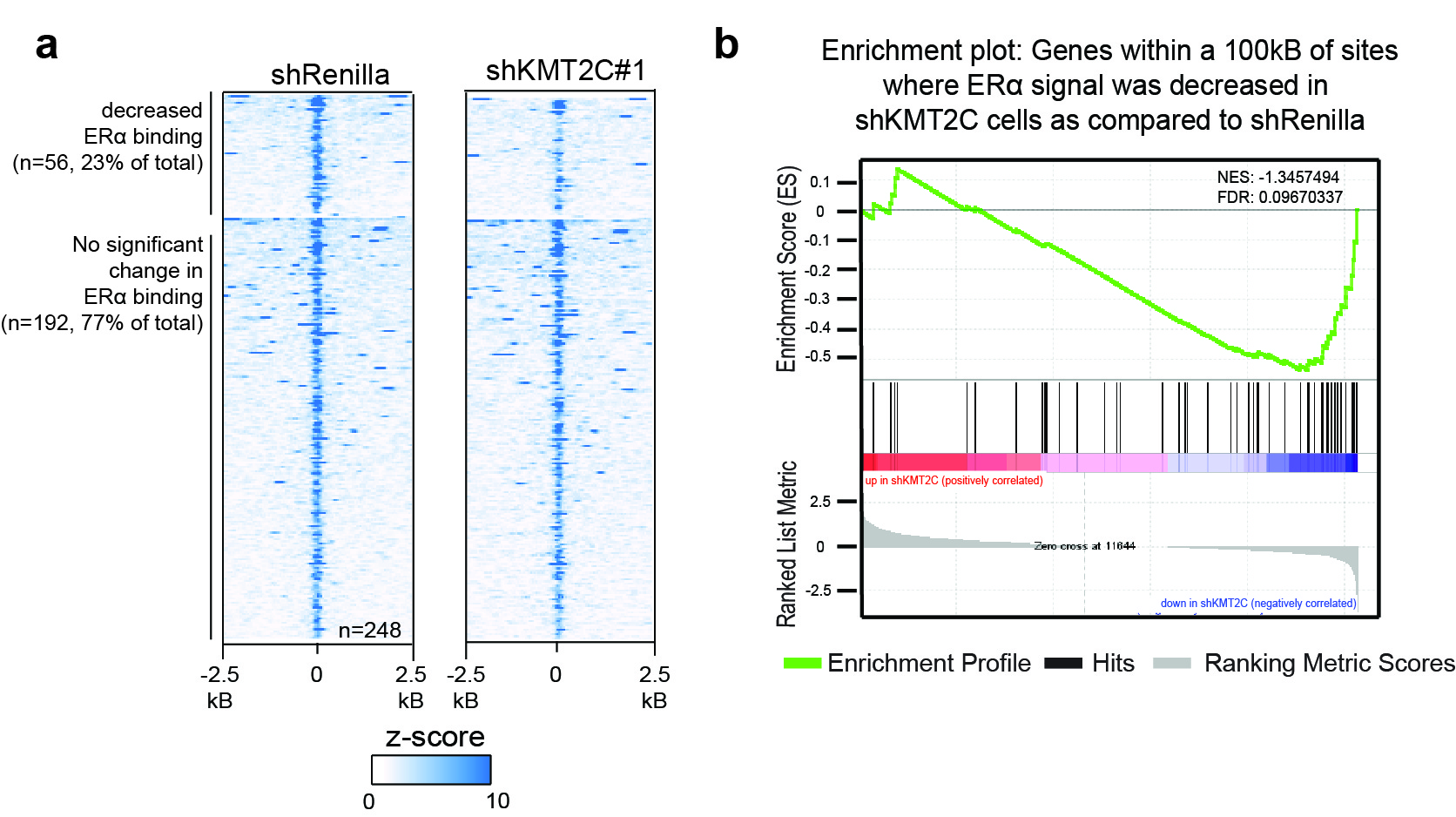


Supplementary Figure 12. Loss of KMT2C does not markedly affect ERα binding to DNA. (A) ERα ChIP Sequencing was performed on MCF7 cells cultured in full serum. Normalized heatmaps for ERα occupancy at sites with decreased H3K4me1 in shKMT2C#1 cells as compared to shRenilla cells. Heatmaps are centered at the peak summit. (B) GSEA of 3857 genes significantly downregulated in MCF7 shKMT2C cells (p-val < 0.05) as compared to ranked genes with a ≥ 25% reduction in ERα marks at enhancers in shKMT2C MCF7 cells.


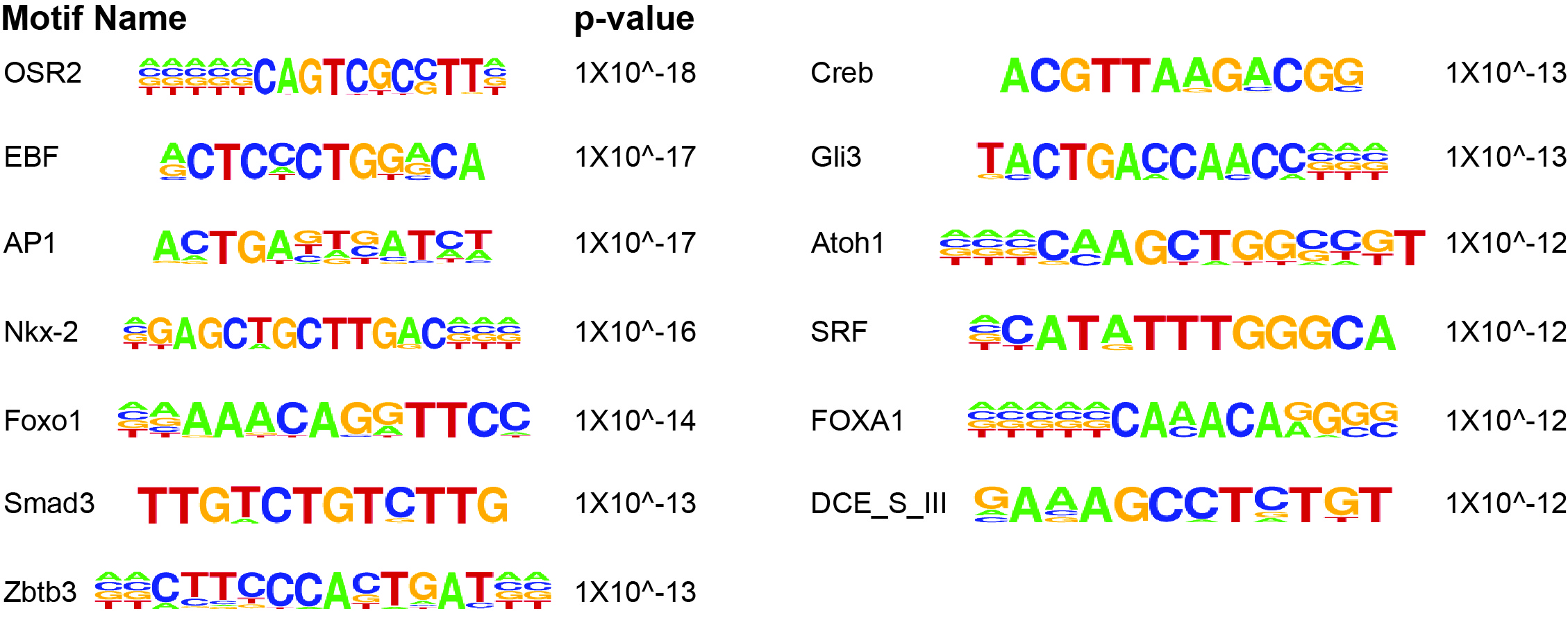


Supplementary Figure 13. Motif analysis, using HOMER, of a 1kB radius around the 869 loci where H3K4me1 enrichment was reduced in shKMT2C#1 and shKMT2C#2 MCF7 cells.


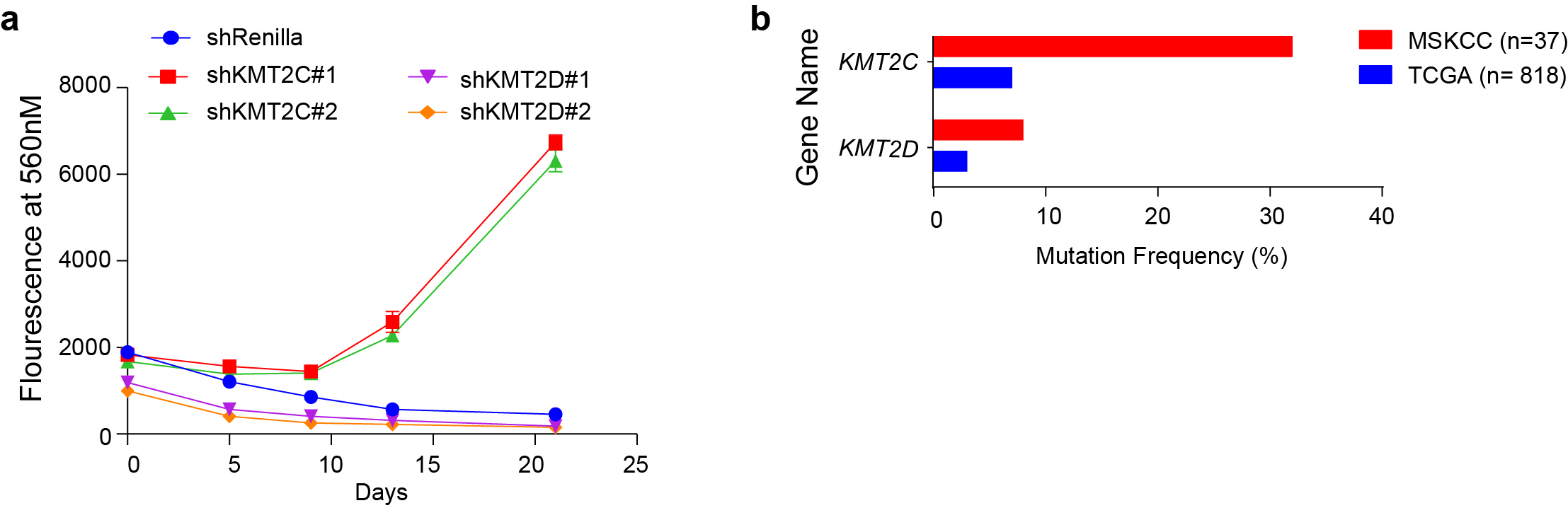


Supplementary Figure 14. KMT2C, not KMT2D promotes rapid outgrowth in hormone deprived media. (A) MCF7 cells stably expressing shRenilla, shKMT2C, or shKMT2D were assayed for proliferation in phenol-red free media with charcoal stripped medium using the alamarBlue cell viability assay. Values correspond to the mean of six experimental replicates ± s.e.m.). Data correspond to one representative assay from a total of two independent assays. (B) Percentage of cases with gene mutations detected in patient samples (MSKCC(1)) were compared to those of TCGA(2).


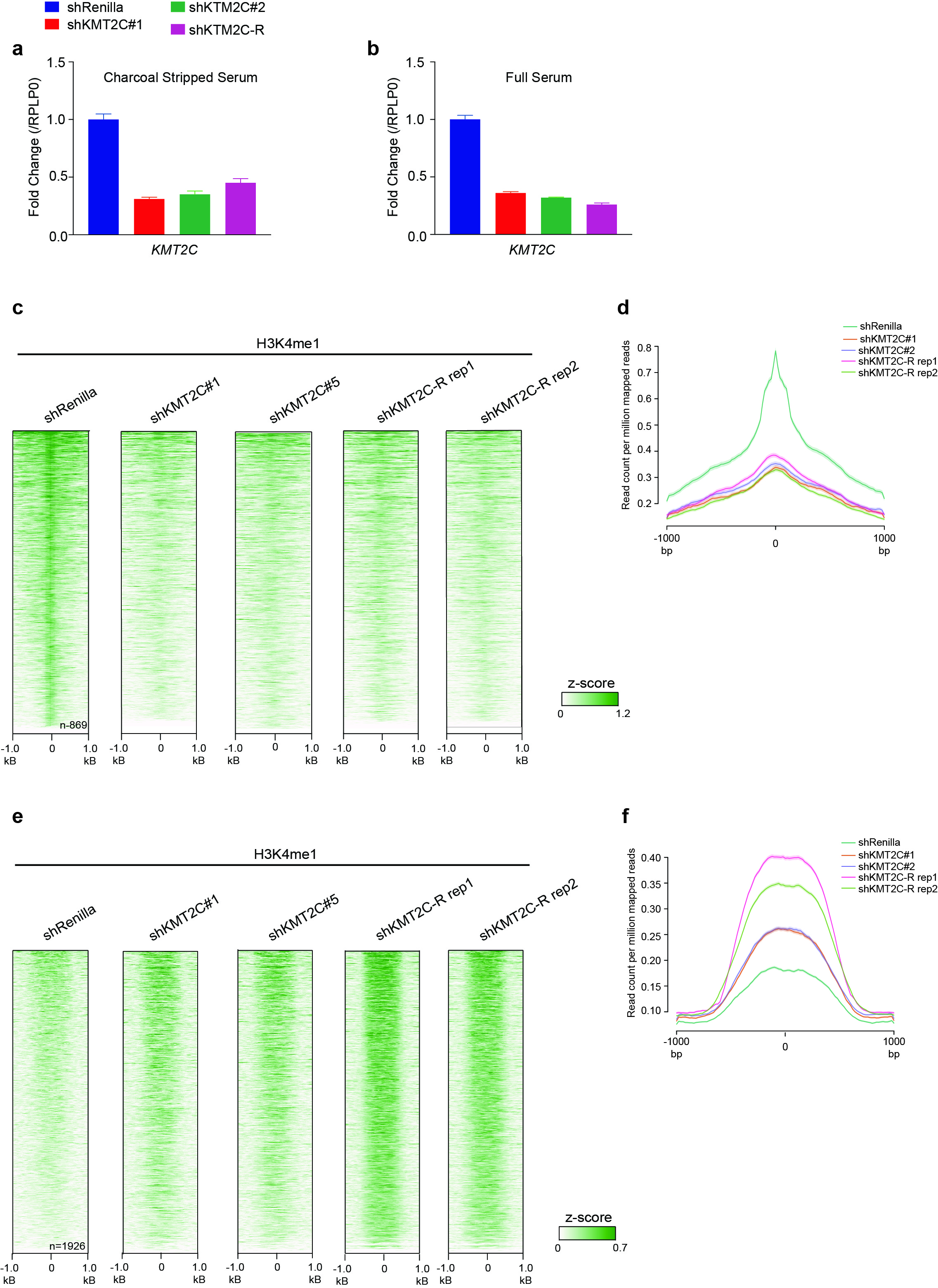


Supplementary Figure 15. H3K4me1 profile in shKMT2C-R cells. (A) mRNA levels of *KMT2C*, as measured by qRT-PCR, in shRenilla, shKMT2C#1, shKMT2C#2 and shKMT2C-R MCF7 cells. Values correspond to the average of three replicates ± s.e.m. shRenilla, shKMT2C#1 and shKMT2C#2 were cultured in charcoal stripped serum for 48 hours prior to collection. shKMT2C-R cells were maintained and collected in charcoal stripped serum. (B) mRNA levels of *KMT2C*, as measured by qRT-PCR, in shRenilla, shKMT2C#1, shKMT2C#2 and shKMT2C-R MCF7 cells. Values correspond to the average of three replicates ± s.e.m. shKMT2C-R was cultured in full serum for 48 hours prior to collection. shRenilla, shKMT2C#1 and shKMT2C#2 cells were maintained and collected in full serum. (C) Normalized heatmaps for H3K4me1 occupancy in shRenilla, shKMT2C#1, shKMT2C#2 and shKMT2C-R MCF7 cells among 869 differential sites where H3K4me1 was reduced in shKMT2C#1, shKMT2C#2 as compared to shRenilla (same sites as depicted in Figure 4). Heatmaps are centered at the peak summit. (D) The average binding of H3K4me1 in shRenilla, shKMT2C#1, shKMT2C#2 and shKMT2C-R MCF7 cells at 869 sites of H3K4me1 loss at a region centered at the peaks and showing ±1kB.


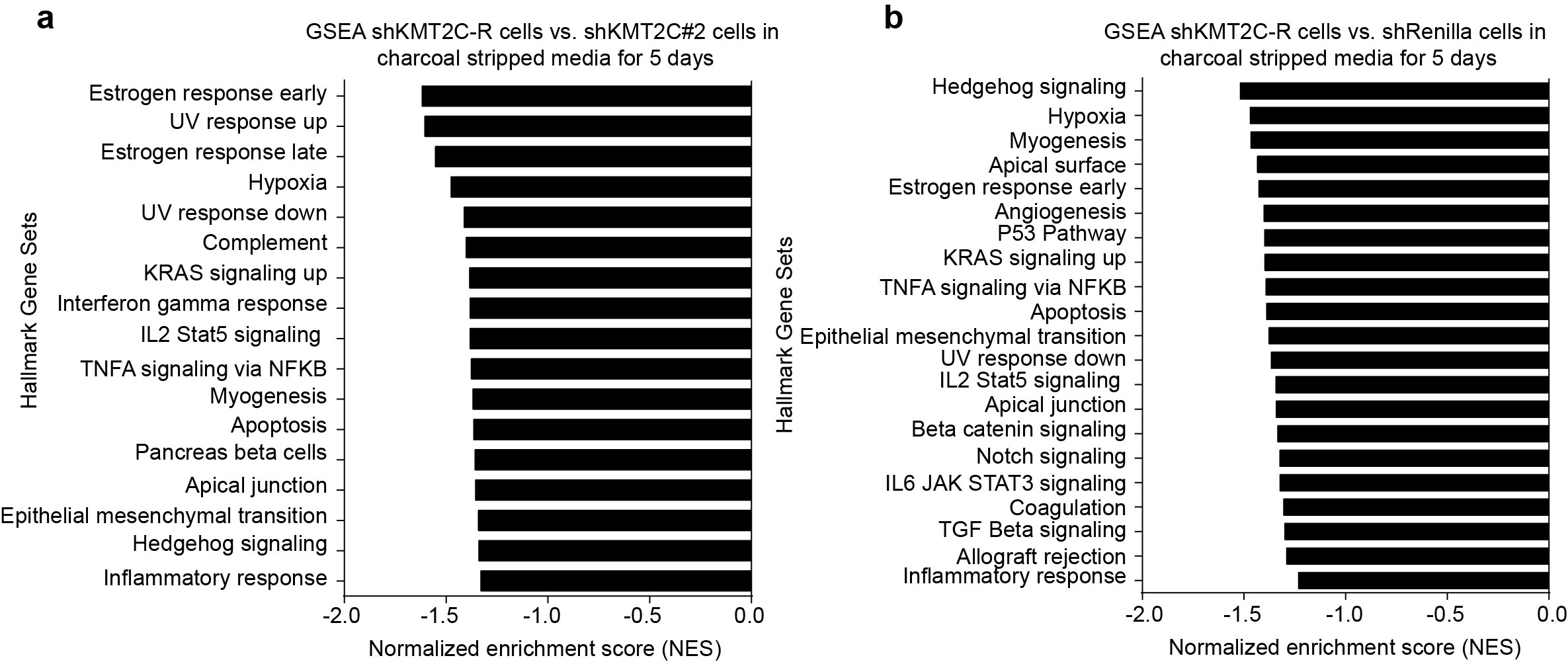


Supplementary Figure 16. Deregulated pathways in shKMT2C-R cells. (A) GSEA of 4347 genes significantly downregulated in MCF7 shKMT2C-R cells as compared to MCF7 shKMT2C cells in charcoal stripped media for 5 days (p-val < 0.05) against Hallmark panel of gene sets (50 gene sets, downloaded from the Broad Institute Molecular Signature Database). Gene sets shown all had an FDR q-value of at least 0.25 (B) GSEA of 4559 genes significantly downregulated in MCF7 shKMT2C-R cells as compared to MCF7 shRenilla cells in charcoal stripped media for 5 days (p-val < 0.05) against Hallmark panel of gene sets (50 gene sets, downloaded from the Broad Institute Molecular Signature Database). Gene sets shown all had an FDR q-value of at least 0.25.

Supplementary Figure 17. Gene expression signatures of KMT2C-R cells correlate with poor progression. (A-C) Molecular stratification of Luminal A and Luminal B patients in the METABRIC cohort using gene expression signatures derived from KMT2C knockdown experiments.  Results are shown for three experimentally derived gene signatures that include genes downregulated in shKMT2C vs. shRenilla (A), genes downregulated in shKMT2C-R vs. shKMT2C#2 in CSS (B) and genes downregulated in shKMT2C-R vs. shRenilla in CSS (C).  Kaplan-Meier curves show overall survival for patients with high signature scores (1st quartile, purple), low signature scores (4th quartile, yellow) and intermediate signature scores (2nd and 3rd quartiles, gray).  Heat maps show normalized gene expression for the genes included in each signature across the 1209 patients, ranked from left to right in increasing order of signature score. Luminal A or B PAM50 subtyping indicated by light or dark blue lines. Columns represent individual patients and rows represent probes. Three-class comparison log-rank p-values for survival curves= 0.0277, 0.0086, 0.0002 for the left, middle and right graphs respectively. Two-class comparison log-rank p-values between high and low scoring patients= 0.0093, 0.0031, 0.00024 for left, middle and right graphs, respectively.


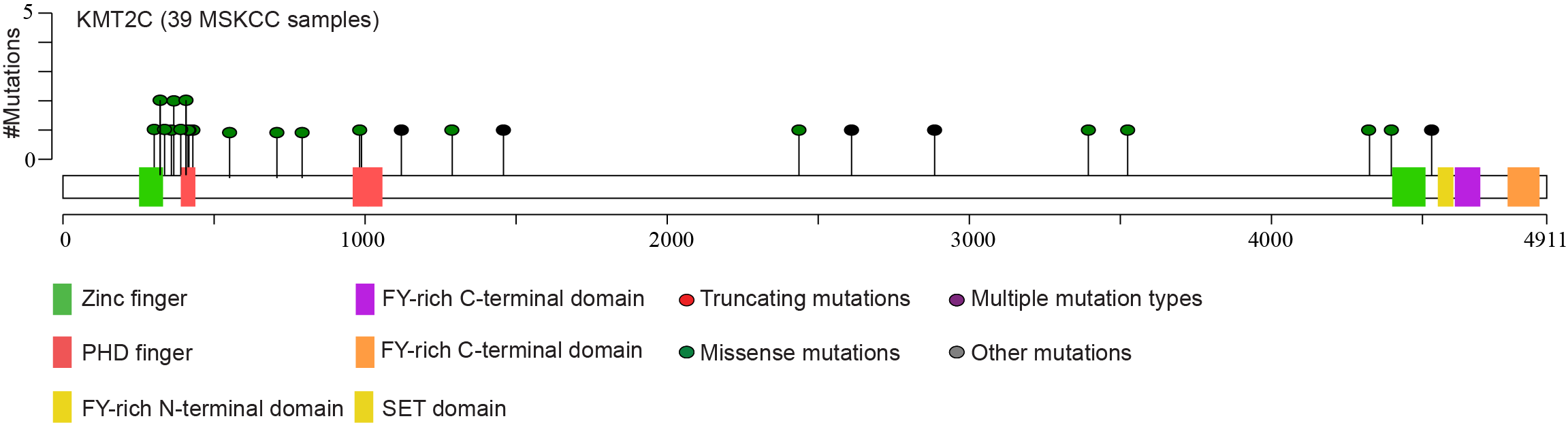


Supplementary Figure 18. Diagram of KMT2C domains with the locations of the identified MSKCC mutations. y-axis corresponds to the number of cases with indicated mutation (n=39).


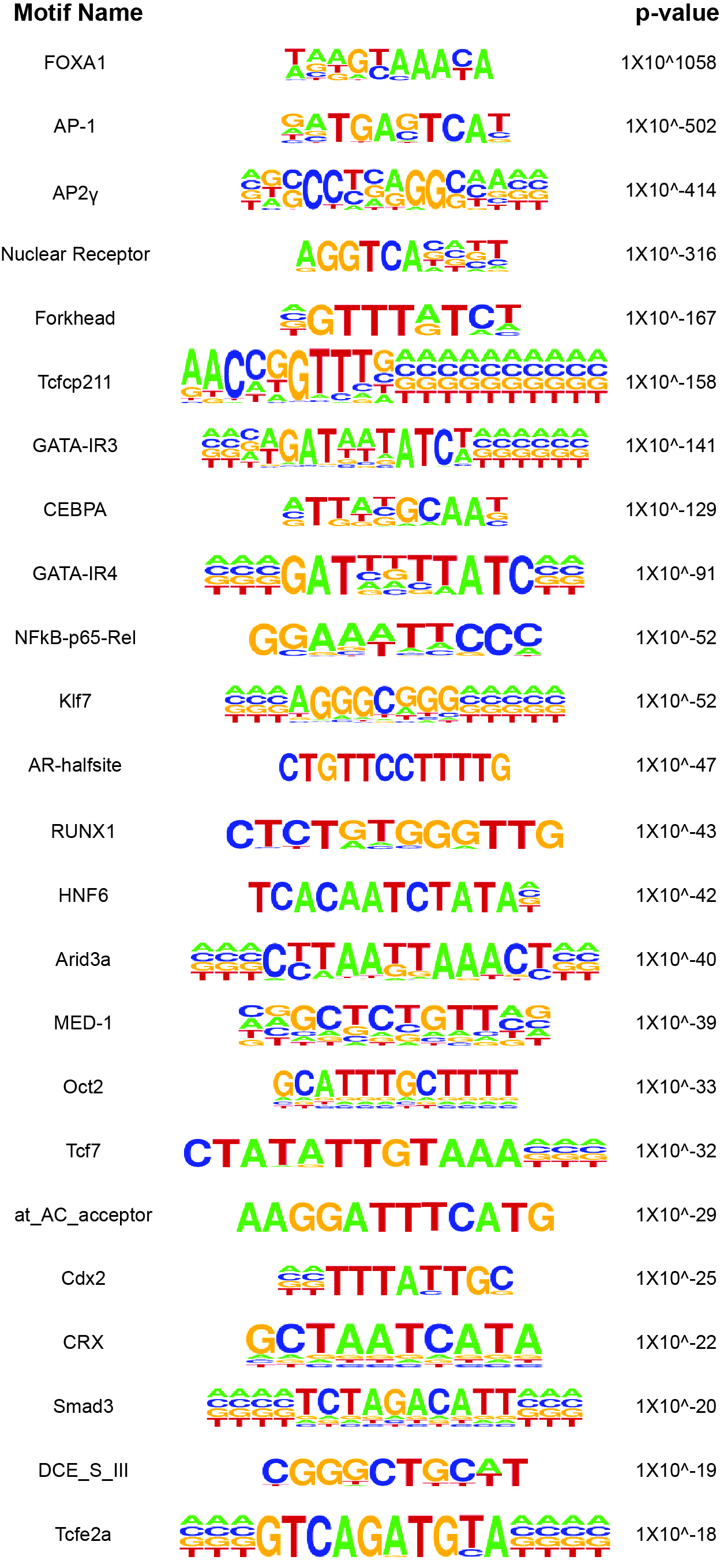


Supplementary Figure 19. Motif analysis, using HOMER, at the 10,512 loci novel ERα loci in shKMT2C-R (maintained in charcoal stripped serum) as compared to shRenilla MCF7 cells (maintained in full serum).

References:

1. Toy W*, et al.* (2013) ESR1 ligand-binding domain mutations in hormone-resistant breast cancer. *Nature genetics* 45(12):1439-1445.

2. Ciriello G*, et al.* (2015) Comprehensive Molecular Portraits of Invasive Lobular Breast Cancer. *Cell* 163(2):506-519.
