# Supplementary material for: KMT2C mediates the estrogen dependence of breast cancer through regulation of ERα enhancer function
Source: Oncogene. 2018 May 14;37(34):4692–710. doi: 10.1038/s41388-018-0273-5 (PMC6107480; doi:10.1038/s41388-018-0273-5)
Supplement: Supplementary file 2 — Supplemental Table 1 [file 41388_2018_273_MOESM2_ESM.docx]

| **Cell Line** | **Chromosome** | **Nucleotide** | **Ref** | **Alt** |
| --- | --- | --- | --- | --- |
| **Cama-1** | ***KMT2C* WT** | | | |
| **HCC-1806** | ***KMT2C WT*** | | | |
| **HCC-1954** | ***KMT2C WT*** | | | |
| **MDA-MB-361** | ***KMT2C* WT** | | | |
| **MCF7** | ***KMT2C* WT** | | | |
| **MDA-MB-231** | ***KMT2C* WT** | | | |
| **MDA-MB-468** | ***KMT2C WT*** | | | |
| **MCF10A** | ***KMT2C* WT** | | | |
| **T47D** | **7** | **151932996** | **C** | **T** |
| **SKBR3** | **7** | **151878785** | **G** | **C** |

Supplementary Table 1. *KMT2C* mutation status in breast cell lines.
