# Supplementary material for: KMT2C mediates the estrogen dependence of breast cancer through regulation of ERα enhancer function
Source: Oncogene. 2018 May 14;37(34):4692–710. doi: 10.1038/s41388-018-0273-5 (PMC6107480; doi:10.1038/s41388-018-0273-5)
Supplement: Supplementary file 4 — Supplemental Table 3 [file 41388_2018_273_MOESM4_ESM.docx]

Supplementary Table 3. IMPACT mutation analysis of shKMT2C#2-R cells

| Gene | Protein Change | Allele Freq. in shKMT2C#2 parental | Allele Freq. in shKMT2C#2 resistant | COSMIC |
| --- | --- | --- | --- | --- |
| ABL1 | **P900L** | 0.06 | not present |  |
| BRD4 | **P956Tfs*137** | 0.15 | not present |  |
| PIK3C2G | **Q404E** | 0.16 | not present |  |
| RPTOR | **R330Q** | not present | 0.19 | 2 |
| KEAP1 | **H451Y** | not present | 0.05 |  |
| PIK3CA | **E545K** | 0.30 | 0.23 | 1431 |
| GATA3 | **D335Gfs*17** | 0.46 | 0.50 | 1 |
| ATRX | **D2136V** | 0.07 | 0.07 | 2 |
| ERBB4 | **Y1242C** | 0.22 | 0.24 | 2 |
| PTPRD | **G61E** | 0.33 | 0.29 | 2 |
| FGF3 | **R120W** | 0.22 | 0.24 | 1 |
| IRS1 | **R327C** | 0.32 | 0.33 | 1 |
| MAP3K13 | **D380N** | 0.23 | 0.24 | 1 |
| CHEK2 | **R145L** | 0.18 | 0.23 |  |
| HIST1H1C | **A24T** | 0.45 | 0.47 |  |
| NBN | **P325H** | 0.09 | 0.10 |  |
| NBN | **R43*** | 0.68 | 0.67 |  |
| PHOX2B | **Y83C** | 0.41 | 0.13 |  |
| SF3B1 | **E870K** | 0.24 | 0.21 |  |
| SPOP | **S330L** | 0.19 | 0.22 |  |
| TBX3 | **P602S** | 0.21 | 0.18 |  |
| ZFHX3 | **Q1740_Q1741del** | 0.25 | 0.24 |  |
